# Trade-Off Relation between Fungicide Sensitivity and Melanin Biosynthesis in Plant Pathogenic Fungi

**DOI:** 10.1016/j.isci.2020.101660

**Published:** 2020-10-08

**Authors:** Ken Harata, Hiroyuki Daimon, Tetsuro Okuno

**Affiliations:** 1 Department of Plant Life Science, Ryukoku University, Seta, Shiga 520-2194, Japan; 2 Graduate School of Agriculture, Ryukoku University, Seta, Shiga 520-2194, Japan

**Keywords:** Genetics, Mycology, Agricultural Science

## Abstract

Circumventing the emergence of fungicide-resistant strains is a crucial issue for robust disease management in agriculture. The agricultural fungicide ferimzone has been used for the control of rice diseases including rice blast. The emergence of ferimzone-resistant strains in rice fields has not been reported. Here, we identified the copper transport *CoICT1* gene as the ferimzone sensitivity gene in *Colletotrichum orbiculare* and the rice blast fungus *Magnaporthe oryzae*. Genetic and cytological analyses showed that functional defects in the copper transport pathways, consisting of CoIct1 and P-type ATPase CoCcc2, led to the low sensitivity to ferimzone and the pathogenicity defect due to attenuated melanization in the appressorium. Importantly, the presence of CuSO_4_ induced high sensitivity to ferimzone even in the *coict1* mutant. Our study shows that there is a trade-off relation between the sensitivity to ferimzone and fungal pathogenicity.

## Highlights

• Fungal copper transporters, Ict1 and Ccc2, are involved in ferimzone sensitivity
• Melanin biosynthesis requires a laccase activity instigated by Ict1-mediated copper
• A metal-binding site in Ict1 is crucial for ferimzone sensitivity and pathogenicity
• CuSO_4_ has an enhancing effect on ferimzone sensitivity

## Graphical Abstract

**Figure:**
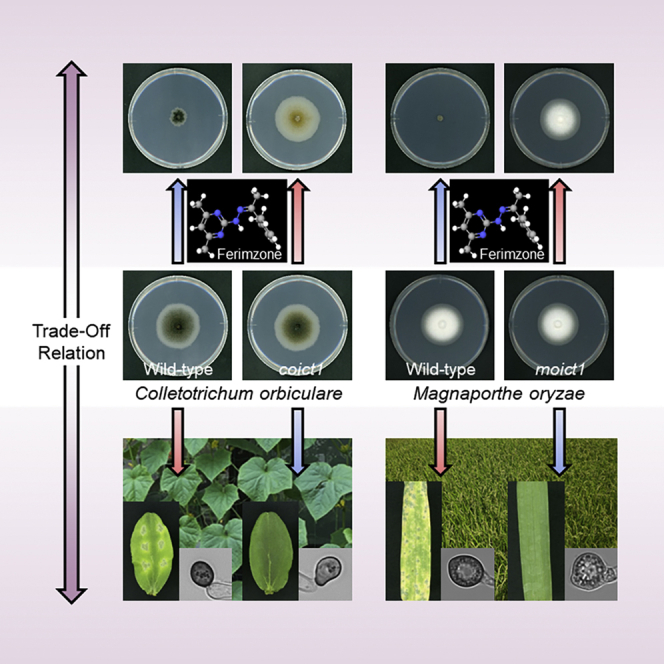

## Introduction

Rice is one of the most important cereal crops and accounts for a major source of calories in Asia, where 60% of the world population lives (Khush, 2005). The rice blast fungus *Magnaporthe oryzae* annually causes 10–30% loss in cultivated rice (Dean et al., 2012). Recently, this pathogen caused a serious disease in wheat in Asia (Islam et al., 2016; Inoue et al., 2017). A diverse array of fungicides has been developed for the control of plant diseases, including rice blast, over the last five decades. The application of these fungicides has been critical to effective disease management in agriculture. However, fungicide-resistant strains have emerged, including strains with resistance to quinone outside inhibitors (QoIs) and inhibitors of scytalone dehydrogenase in melanin biosynthesis (Takagaki et al., 2004; Castroagudín et al., 2015). At present, the main countermeasure to circumvent the emergence of fungicide-resistant strains is the rotational or combined use of two or more fungicides with different modes of action. Therefore, there is need for a strategy against fungicide-resistant strains in order to maintain the current level of robust disease control in agriculture.

Ferimzone is a fungicide used for the control of rice diseases, particularly rice blast disease caused by *M. oryzae.* Our knowledge about the mode of action of ferimzone is quite limited. Ferimzone does not affect the respiratory activity of the mycelia of *M. oryzae,* and its antifungal activity is fungistatic (Okuno et al., 1989a). Ferimzone causes the leakage of some electrolytes from mycelia and inhibits the uptake of sodium acetate from an incubation medium, suggesting that ferimzone affects membrane function (Okuno et al., 1989a, 1989b). Ferimzone became commercially available in agricultural fields in 1993. Interestingly, since then, the emergence of ferimzone-resistant *M. oryzae* strains have not been reported. How ferimzone circumvents a pandemic by resistant strains, if any, in the field remains elusive. This feature of ferimzone prompted us to study its mode of action in detail.

*Colletotrichum orbiculare* causes anthracnose disease in cucurbitaceous plants. Like *M. oryzae,* this fungus develops a dome-shaped cell, called an appressorium, at the tip of a germ tube (Kubo and Takano, 2013). Melanin pigment, which is biosynthesized from a starter metabolite, acetyl CoA, is accumulated on the appressorial cell wall (Kubo et al., 1991; Takano et al., 1995). The melanin layer is a semi-permeable membrane that prevents efflux of cellular glycerol and permits the generation of high turgor pressure to penetrate the tough cuticle layer and cell walls of plants. Therefore, appropriate melanin biosynthesis is a crucial step for appressorium-mediated infection in both *M. oryzae* and *C. orbiculare*. In *Colletotrichum lindemuthianum* and *Botrytis cinerea*, copper-transporting ATPase *ClAP1* and *BcCcc2* are involved in melanization and pathogenicity (Parisot et al., 2002; Saitoh et al., 2010). In *C. orbiculare*, a laccase gene (*CoLAC2*) encoding a protein with multicopper oxidation domains participates in the final step of melanin biosynthesis, in which 1,8-dihydroxynaphthalene is oxidized (Lin et al., 2012). These reports suggest that intracellular copper ions are involved in the melanization and pathogenicity of phytopathogenic fungi. However, the molecular mechanisms underlying copper ion transport to the laccase remain unclear.

**Figure 1:**
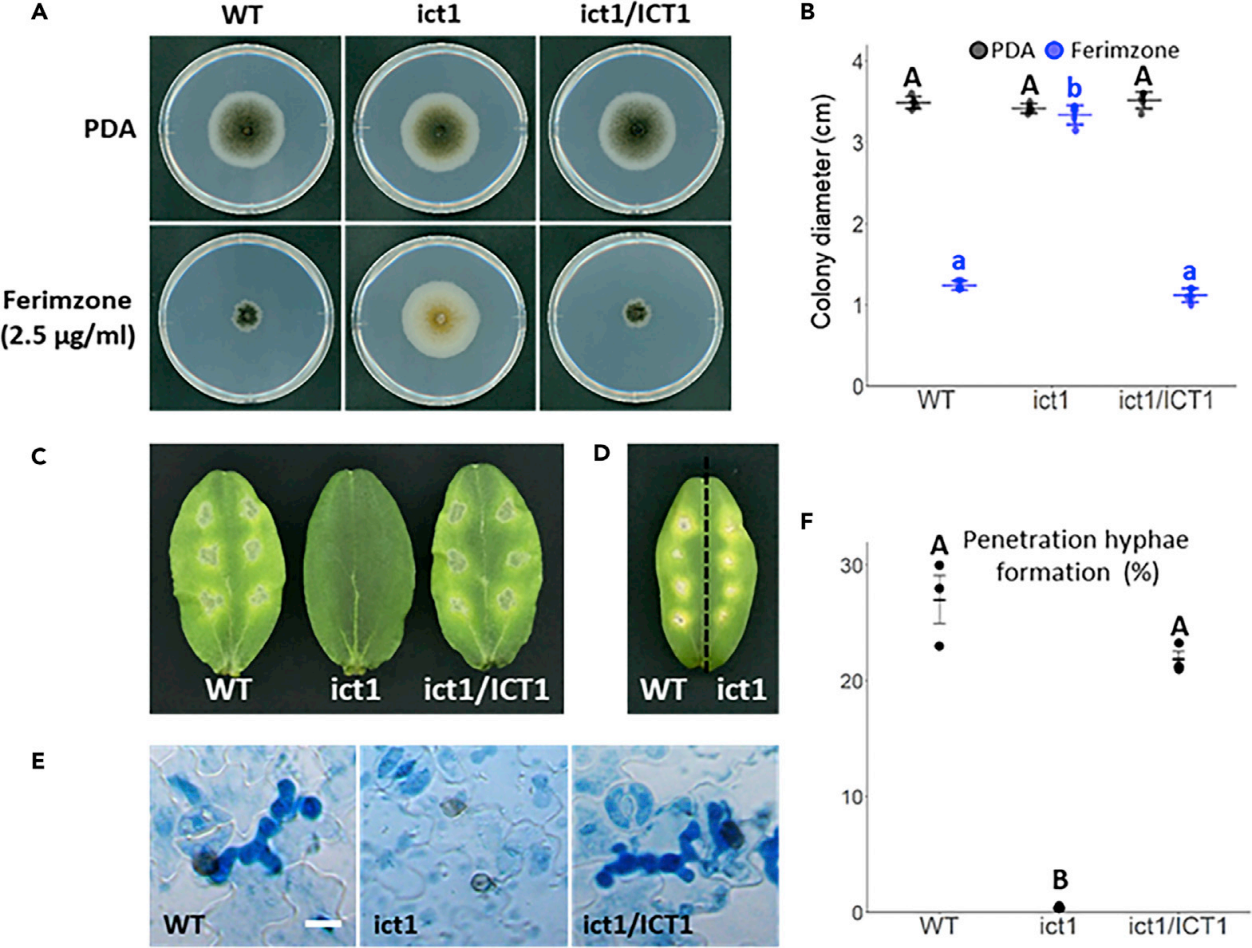
The *coict1* Mutant Shows a Low Sensitivity to Ferimzone and Pathogenesis on Cucumber Leaves (A) Ferimzone-sensitivity tests of the *coict1* mutant. A mycelia block of each strain was placed on ferimzone-containing PDA medium, respectively, and was incubated for six days at 24°C. WT, wild-type; ict1, *coict1* mutant; ict1/ICT1, *CoICT1*-complemented transformant. (B) Average of a colony diameter of the *coict1* mutant on the ferimzone-containing PDA medium. Error bars represent the standard deviation of the mean (n = 5). Different letters above the scatter plots of each column represent significant differences (Tukey's HSD test; p < 0.001). (C) Pathogenicity assays on intact cucumber cotyledons. Conidial suspensions of each strain were dropped onto the adaxial surface of cucumber cotyledons and inoculated leaves were incubated for six days at 24°C. (D) Pathogenicity assays on wounded cucumber cotyledons. Conidial suspensions of each strain were dropped onto the wounded sites of cucumber cotyledons and inoculated leaves were incubated for six days at 24°C. (E) Penetration hyphae formation of the *coict1* mutant on cucumber cotyledons. Conidial suspensions of each strain were dropped onto the abaxial surface of cucumber cotyledons and inoculated leaves were incubated for three days at 24°C. Scale bar, 10 μm. (F) Percentage of appressorium-mediated penetration hyphae formation in the *coict1* mutant. Approximately, 300 appressoria were observed per one experiment. Three independent experiments were conducted, and error bars represent standard deviation of the mean. Different letters above the scatter plots of each column represent significant differences (Tukey's HSD test; p < 0.001).

In this study, to elucidate the molecular mechanisms, if any, underlying the circumvention of occurrence of ferimzone-resistant *M. oryzae* strains in fields, first we searched for candidate genes involved in ferimzome sensitivity using *C. orbiculare* transfer DNA insertion mutants because *C. orbiculare* like *M. oryzae* is sensitive to ferimzone (Figure S1. Average Colony Diameter in the Wild-type on the PDA Medium Containing Different Concentrations of Ferimzone, Related to Figures 1 and 6) and because more than 90% transfer DNA insertion lines generated by *Agrobacterium tumefaciens*-mediated transformation have a single copy insertion (Tsuji et al., 2003). Moreover, *C. orbiculare* and *M. oryzae* deploy common infection processes such as infection-related morphogenesis and melanin biosynthesis. Thus, *C. orbiculare* is suitable for an alternative model fungus of *M. oryzae* to identify and analyze genes involved in ferimzone sensitivity. We screened *C. orbiculare* mutants with low sensitivity to ferimzone from its transfer DNA insertion libraries and identified a heavy metal-associated domain-encoding gene, *CoICT1*, as a candidate gene involved in the sensitivity to ferimzone. By genetic and cytological analyses using gene disruption mutants of *C. orbiculare*, we found that a functional loss of copper transport pathways, consisting of CoIct1 and P-type ATPase CoCcc2, led to the low sensitivity to ferimzone and an attenuated melanization in the appressorium. Moreover and importantly, we revealed that these phenotypes are also conserved in *M. oryzae*. Taken together, these results show that there is a trade-off relation between the sensitivity to ferimzone and the pathogenicity of fungi in the copper transport pathway.

## Results

### *CoICT1* Is Involved in Sensitivity to Ferimzone and Appressorial Melanization

We previously generated 6996 random transfer DNA insertion lines in *C. orbiculare* (Harata and Okuno, 2019). To isolate a mutant with low sensitivity to ferimzone, we observed the colony growth of T-DNA-inserted transformants on potato dextrose agar (PDA) medium containing ferimzone at the concentration of 10 μg/mL, which inhibits colony growth of the wild-type (Figure S1). Through this screening, we obtained a ferimzone-tolerant transformant 1 (FT1) (Figure S2. The FT1 Transformant Shows a Low Sensitivity to Ferimzone, Related to Figure 1) and examined the mutated gene by thermal asymmetric interlaced (TAIL) PCR.

**Figure 2:**
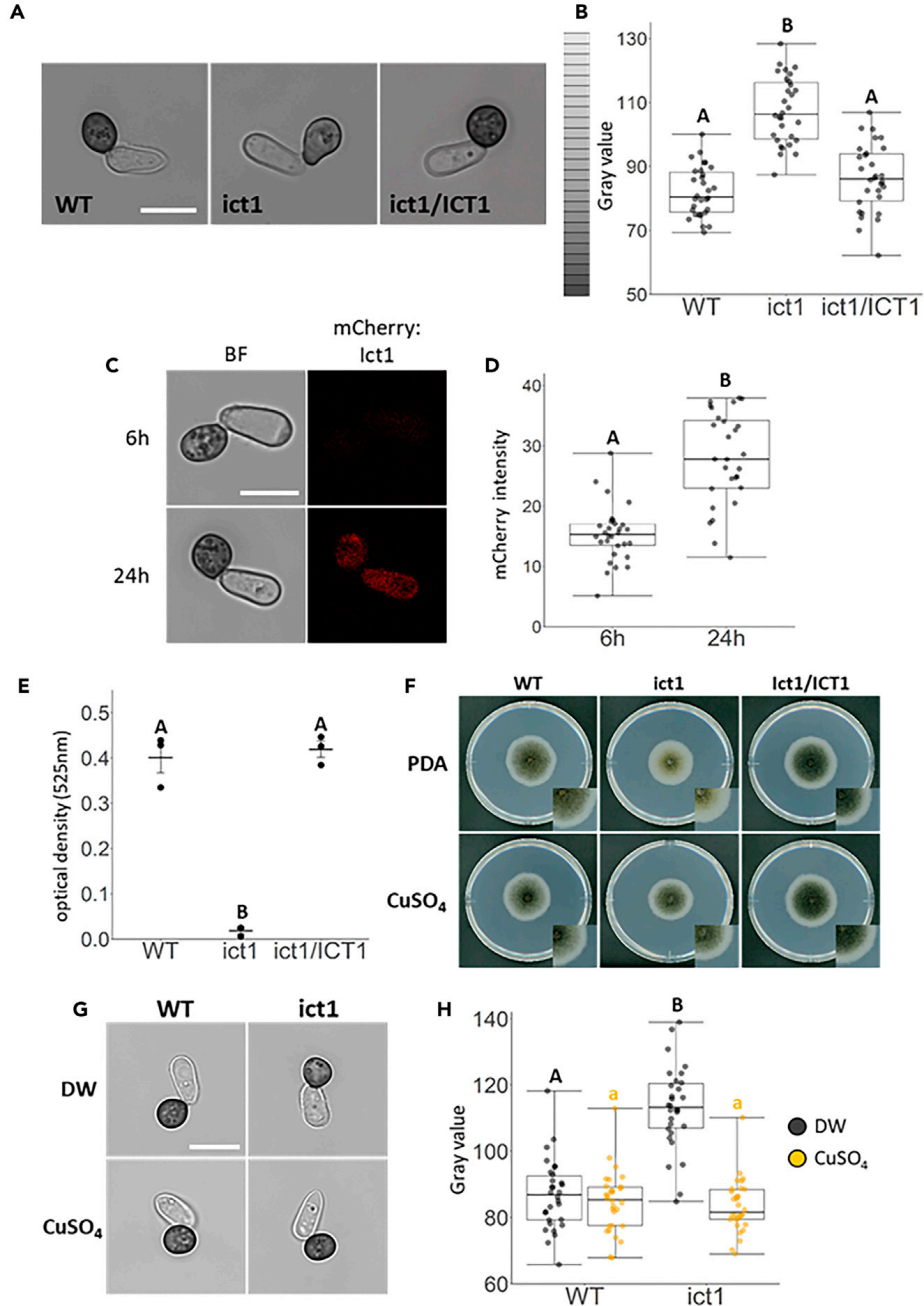
CoIct1 is Involved in Appressorial Melanization through the Cu Transport Required for Laccase Activity (A) Observation of appressorial melanization in the *coict1* mutant. Conidial suspensions of each strain were placed on cover slips and incubated for 24 h at 24°C. WT, wild-type; ict1, *coict1* mutant; ict1/ICT1, *CoICT1*-complemented transformant. Scale bar, 10 μm. (B) Gray scale value of appressorial melanization in the *coict1* mutant. Approximately 30 appressoria of each strain were measured. Different letters above the box plots represent significant differences (Tukey's HSD test; p < 0.001). (C) Subcellular localization of mCherry-CoIct1 during appressorial melanization. Conidial suspensions of each strain were placed on cover slips and incubated for 6 h or 24 h at 24°C. Scale bar, 10 μm. (D) Signal intensity of mCherry-Ict1 in an appressorial cell. Approximately, 30 appressoria were measured. Different letters above the box plots represent significant differences (Wilcoxon signed-rank test; p < 0.001). (E) Laccase activity in the *coict1* mutant. Laccase activity was measured in the culture filtrate of each strain. Three independent experiments were conducted, and error bars represent standard deviation of the mean. Different letters above the scatter plots of each column represent significant differences (Tukey's HSD test; p < 0.001). (F) Melanin color in a colony of the *coict1* mutant on CuSO_4_-supplemented PDA media. A mycelia block of each strain was placed on 10 μM CuSO_4_-supplemented PDA medium, respectively, and was incubated for six days at 24°C. (G) Appressorial melanization of the *coict1* mutant in the presence of CuSO_4_. Conidia suspended in distilled water (DW) or 10 μM CuSO_4_ solution were placed on cover slips and incubated for 24 h at 24°C. Scale bar, 10 μm. (H) Gray scale value of appressorial melanization of the *coict1* mutant in the presence of CuSO_4_. Approximately, 30 appressoria of each strain were measured. Different letters above the box plots represent significant differences (Student t-test; p < 0.001).

Sequence analysis of the amplified products of FT1 DNA by TAIL PCR indicated that transfer DNA was inserted into the open reading frame region of Cob-11716, which encodes a 84 amino acid long protein comprising a heavy metal-associated domain. In a BLASTp search, the amino acid sequence of Cob_11,716 showed high homology with those of iron copper transporter-related genes in *A. fumigatus* and *S. cerevisiae*, and we named this gene *CoICT1* (iron copper transporter) (Figure S3. The Amino Acid Sequence of CoIct1 Shows High Homology with Atx1 of A. fumigatus and S. cerevisiae, Related to Figure 1). To examine whether *CoICT1* is involved in the sensitivity to ferimzone, we generated *coict1* mutants and observed their hyphal growth in the presence of ferimzone. Disruption of the targeted gene was confirmed by Southern blot analysis (Figure S4A. Confirmation of Targeted Gene Disruptions by Southern Blotting Analyses, Related to Figure 1). The colony diameter of the *coict1* mutant was much larger than that of the wild-type or *CoICT1*-complemented transformant on PDA medium containing ferimzone (Figures 1A and 1B), indicating that the low sensitivity of the *coict1* mutant to ferimzone is acquired by the loss of *CoICT1* function in *C. orbiculare*. To examine whether the *coict1* mutant is able to cause disease symptoms on host plants, we inoculated intact cucumber cotyledons with the spores of *coict1* mutant produced on PDA medium. The *coict1* mutant was unable to form lesions on the intact leaves, suggesting that *CoICT1* is required for fungal pathogenicity (Figure 1C). We also performed a pathogenicity test of the *coict1* mutant on wounded leaves. The *coict1* mutant had the ability to cause disease symptoms on the wounded sites of leaves (Figure 1D). Next, we examined whether the *coict1* mutant could develop appressorium-mediated penetration hyphae inside epidermal cells. The frequency of penetration hyphae was significantly lower in the *coict1* mutant than in the wild-type and *CoICT1*-complemented transformant (Figures 1E and 1F). Importantly, the melanin pigmentation of appressoria in the *coict1* mutant appeared to be weaker than that in the wild-type or *CoICT1*-complemented transformant (Figure 1E). Consistent with these observations on host epidermal cells, the *coict1* mutant formed a weakly pigmented appressorium on the cover slips (Figures 2A and 2B).

Next, to examine CoIct1 localization, we generated an mCherry:CoICT1 construct that expresses N-terminally *mCherry*-fused *CoICT1* under the native promoter and introduced it into the *coict1* mutant. We found that mCherry:CoICT1 complemented pathogenesis in the introduced transformant on cucumber leaves, indicating that mCherry:CoIct1 is functional (Figure S5A. Pathogenicity Assays on mCherry:ICT1-Introduced Transformant and ICT1:3XFLAG-Introduced Transformant, Related to Figure 2). mCherry fluorescent signals were strongly detected in melanized appressoria but were much weaker in non-melanized appressoria (Figures 2C and 2D). These results demonstrated that *CoICT1* plays a pivotal role in appressorial melanization.

### *CoICT1* Is Required for the Laccase Activity Responsible for Melanization

A laccase-encoding *CoLAC2* gene participates in oxidation of 1,8-dihydroxynaphtalene in the final step of melanin biosynthesis process and is required for the melanization of appressoria and mycelia in *C. orbiculare* (Lin et al., 2012). Copper is known to serve as a cofactor of laccase in fungi. To examine whether *CoICT1* is involved in melanin biosynthesis via laccase activity, we first measured the activity of extracellular laccase using culture filtrates of the *coict1* mutant. The laccase activity of the *coict1* mutant was significantly lower than that of the wild-type and the *CoICT1*-complemented transformant (Figure 2E). Next, we observed colony and appressorial melanization of the *coict1* mutant in the presence of Cu. An exogenous supply of 10 μM CuSO_4_ restored melanin pigmentation in the colony and appressorium of the *coict1* mutant to the levels of the wild-type (Figures 2F–2H). These results suggested that CoIct1 plays a role in melanin biosynthesis through the copper transport required for the laccase activity. To examine whether the exogeneous supply of CuSO_4_ restores pathogenesis of the *coict1* mutant, we inoculated cucumber cotyledons with CuSO_4_-treated spores of the *coict1* mutant. Both strains of the *coict1* mutant and the wild-type did not cause lesions on the cucumber cotyledons (Figure S6. Pathogenicity Assays on CuSO_4_-treated wild-type and coict1 mutant, Related to Figure 2), indicating that the exogeneous CuSO_4_ affected the pathogenicity of *C. orbiculare*. To examine whether a defect of melanin biosynthesis is associated with the low sensitivity to ferimzone, we performed ferimzone-sensitivity tests using disruption mutants of melanin biosynthesis genes, *CoPKS1* polyketide synthase, *CoSCD1* scytalone dehydratase, *CoTHR1* trihydroxynaphthalene reductase, and *CoLAC2* laccase2 (Takano et al., 1995; Lin et al., 2012; Kubo et al., 1996; Perpetua et al., 1996). Colony diameters of all these mutants were almost comparable to that of the wild-type on the PDA medium, although those of the *cothr1* and *colac2* mutants were smaller by 0.2 cm than that of the wild-type on the PDA medium (Figure S7. Ferimzone-Sensitivity Tests in Mutants of Melanin Biosynthesis Genes, Related to Figure 2). Colony diameters of all these mutants except for the *coscd1* mutant were comparable to that of the wild-type on the ferimzone-containing PDA medium (Figure S7). The *coscd1* mutant formed a smaller colony than the wild-type on the PDA medium with and without ferimzone. These results indicated that the melanin biosynthesis pathway has no link to ferimzone sensitivity.

### The *coict1* Mutant Shows High Sensitivity to Ferimzone in the Presence of CuSO_4_

**Figure 3:**
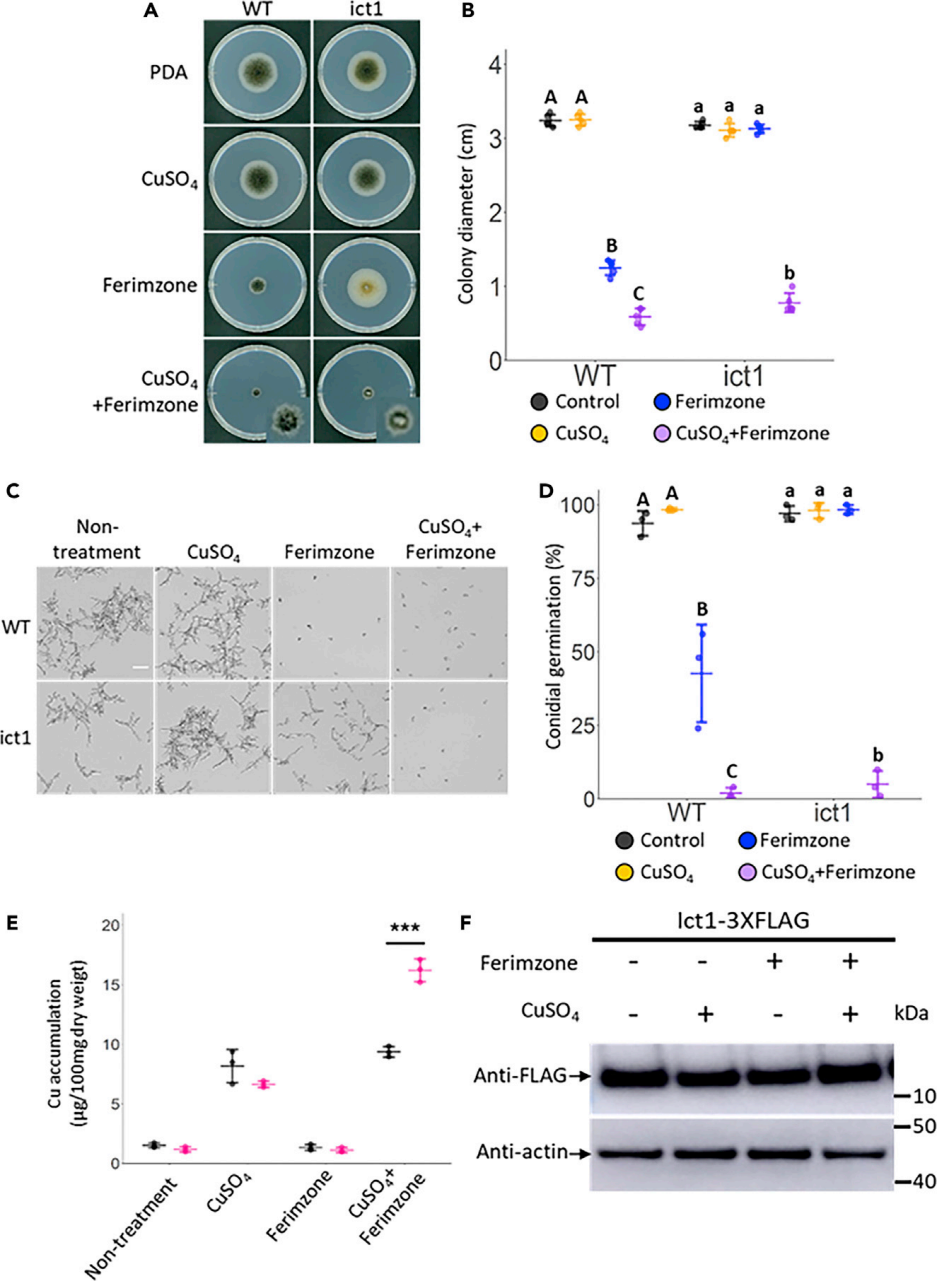
The *coict1* Mutant Shows High Sensitivity to Ferimzone in the Presence of CuSO_4_ (A) CuSO_4_- and ferimzone-sensitivity tests in the *coict1* mutant. A mycelia block of each strain was placed on PDA medium containing CuSO_4_, ferimzone, or both, and was incubated for six days at 24°C. WT, wild-type; ict1, *coict1* mutant. (B) Average colony diameter in the *coict1* mutant on the PDA medium containing CuSO_4_, ferimzone, or both. Error bars represent standard deviation of the mean (n = 5). Different letters above the scatter plots of each column represent significant differences (Tukey's HSD test; p < 0.001). (C) Vegetative hyphae in conidia of the *coict1* mutant treated with CuSO_4_, ferimzone, or both. Conidia of each strain suspended in liquid PSY medium containing CuSO_4_, ferimzone, or both were placed on cover slips and incubated for 24 h at 24°C. Scale bar, 50 μm. (D) Percentage of conidial germination in the *coict1* mutant treated with CuSO_4_, ferimzone, or both. Approximately 100 conidia were observed per one experiment. Three independent experiments were conducted, and error bars represent standard deviation of the mean. Different letters above the scatter plots of each column represent significant differences in the wild-type (Steel-Dwass; p < 0.001) and the *coict1* mutant (Tukey's HSD test; p < 0.001), respectively. (E) Intracellular Cu amount in vegetative hyphae of the *coict1* mutant. The vegetative mycelia treated with CuSO_4_, ferimzone, or both were freeze-dried and then these samples were digested with nitric acid. The Cu amounts in the acid-digested samples were determined by ICP-OES. Error bars represent standard deviation of the mean (n = 3). The asterisk represents a significant difference between the wild-type and the *coict1* mutant (Student's *t*-test: ∗∗∗p < 0.001). (F) CoIct1 expression levels in response to treatment with CuSO_4_, ferimzone, or both. Proteins extractions from mycelia of the *CoICT1*-3X*FLAG* transformant treated with CuSO_4_, ferimzone, or both were conducted by Western blot analysis using anti-FLAG and anti-actin antibody.

Since the *coict1* mutant recovered the ability to form dark colonies in the manner of the wild-type in the presence of CuSO_4_, we tested whether CuSO_4_ could also restore melanization in the *coict1* mutant in the presence of ferimzone, in which the mutant formed albino colonies (Figures 1A and 3A). To examine this possibility, the mycelial block of the *coict1* mutant was incubated on PDA medium containing both ferimzone and CuSO_4_. The *coict1* mutant formed melanized-colonies, but the colony size was much smaller than that on PDA containing ferimzone alone (Figures 3A and 3B), indicating that CuSO_4_ supplementation increased the ferimzone sensitivity of the *coict1* mutant.

Conidia of *C. orbiculare* germinated and developed growth without appressorium differentiation in incubation in liquid PSY at 28°C (Figure 3C). We observed hyphal growth of the *coict1* mutant under this nutrient condition in the presence of ferimzone, CuSO_4_, or both. In the presence of ferimzone, the wild-type halted vegetative hyphal growth, whereas the *coict1* mutant developed vegetative hyphae without branches (Figure 3C). CuSO_4_ had little effect on the hyphal growth of either the wild-type or the *coict1* mutant. However, CuSO_4_ enhanced the susceptibility to ferimzone in both the wild-type and the *coict1* mutant: the combined presence of ferimzone and CuSO_4_ had strong inhibitory effects on the development of vegetative hyphae from conidia and conidial germination in both the wild-type and the *coict1* mutant (Figures 3C and 3D).

In fungi, copper homeostasis is controlled by copper chaperones which act as regulators of the influx and efflux of copper ions in response to various environmental conditions (Smith et al., 2017). We measured the amounts of intracellular Cu in the vegetative hyphae of the *C. orbiculare* wild-type and *coict1* mutant incubated with CuSO_4_, ferimzone, or both, using an inductively coupled plasma optical emission spectrometer (ICP-OES). The amount of Cu in the *coict1* mutant was similar to that in the wild-type and the exogenous supply of CuSO_4_ increased the amount of Cu by approximately four-fold in both the wild-type and the *coict1* mutant (Figure 3E). Ferimzone did not affect the amount of Cu in the wild-type and the *coict1* mutant (Figure 3E). Interestingly, however, the combined presence of ferimzone and CuSO_4_ increased the amount of Cu by approximately two-fold in the *coict1* mutant compared with that in the wild-type (Figure 3E). These findings indicated that the combined presence of ferimzone and CuSO_4_ affected the amount of Cu specifically in the *coict1* mutant. To examine whether CoIct1 expression is altered in response to CuSO_4_, ferimzone, or both, we generated a *CoICT1*:3×*FLAG* construct that expresses C-terminally 3×*FLAG*-fused *CoICT1* under the native promoter and introduced it into the *coict1* mutant. The *CoICT1*:3×*FLAG* introduced-transformant induced lesions similar to those induced by the wild-type on cucumber cotyledons, indicating that this fusion protein is functional (Figure S5B. Pathogenicity Assays on mCherry:ICT1-Introduced Transformant and ICT1:3XFLAG-Introduced Transformant, Related to Figure 3). Western blot analysis showed that the CoIct1 expression levels were not affected by ferimzone, CuSO_4_, or both (Figure 3F).

### Methionine and Cysteine Residues in the MXCXXC Motif of CoIct1 Were Important for Ferimzone Sensitivity and Pathogenicity

**Figure 4:**
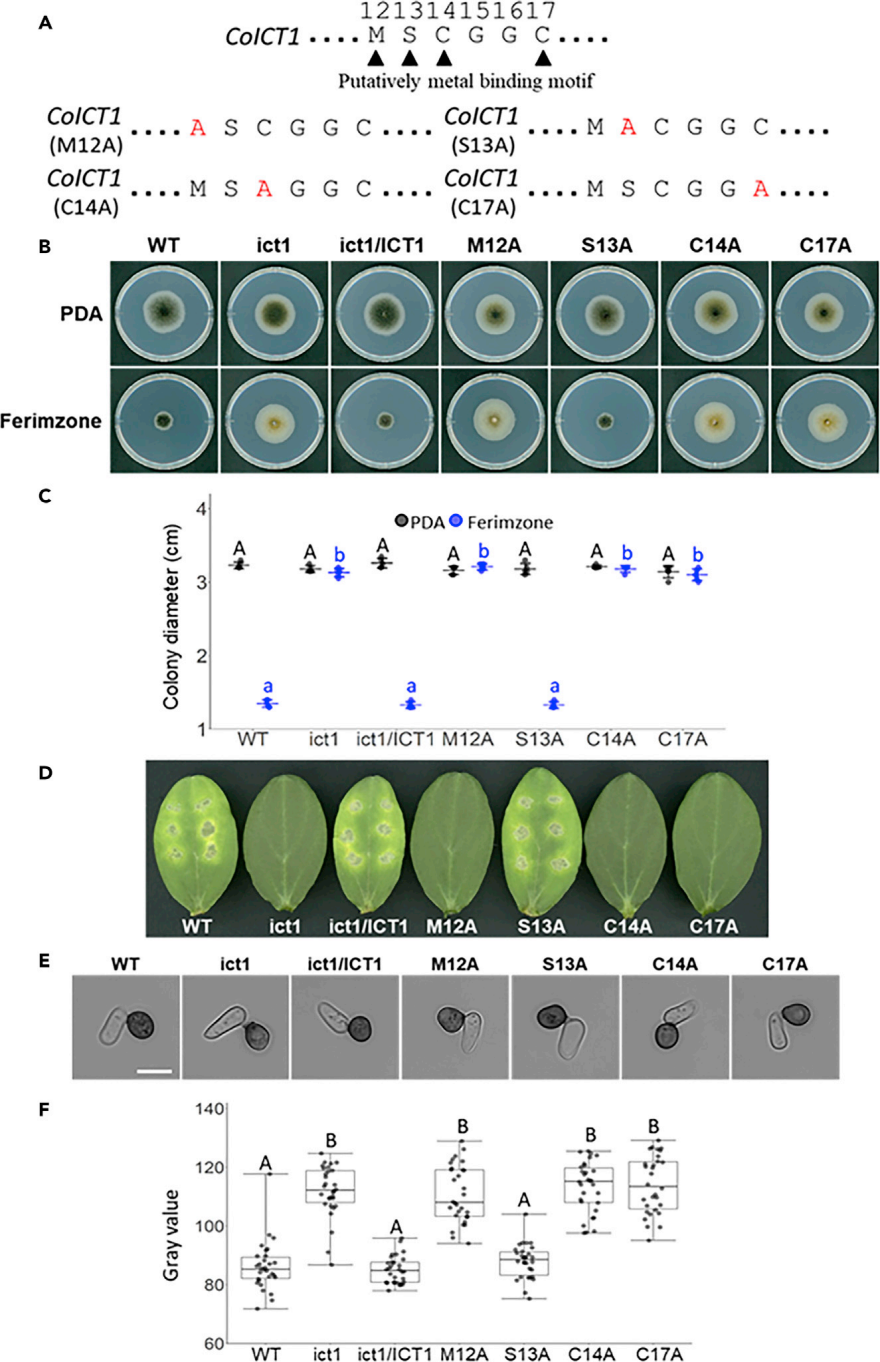
Methionine and Cysteine Residues in the Metal-Binding Motif of CoIct1 Are Important Sites for Ferimzone Sensitivity and Pathogenicity on Cucumber Leaves (A) Amino acid sequences in the putative metal-binding motif of CoIct1. A point mutation site substituted with an alanine residue is indicated in red. (B) Ferimzone-sensitivity tests in the *CoICT1* alanine-scanning transformants. A mycelia block of each strain was placed on the PDA medium containing ferimzone and was incubated for six days at 24°C. (C) Average colony diameter in the *CoICT1* alanine-scanning transformants on the PDA medium containing ferimzone. Error bars represent standard deviation of the mean (n = 5). Different letters above the scatter plots of each column represent significant differences (Tukey's HSD test; p < 0.001). (D) Pathogenicity assays on intact cucumber cotyledons. Conidial suspensions of each strain were dropped onto the adaxial surface of cucumber cotyledons and inoculated leaves were incubated for six days at 24°C. (E) Appressorial melanization of the *CoICT1* alanine-scanning transformants. Conidial suspensions of each strain were placed on cover slips and incubated for 24 h at 24°C. Scale bar, 10 μm. (F) Gray scale value of appressorial melanization of the *CoICT1* alanine-scanning transformants. Approximately, 30 appressoria of each strain were measured. Different letters above the scatter plots of each column represent significant differences (Steel-Dwass test; p < 0.001).

The MXCXXC motif of copper chaperones, which is widely conserved in eukaryotes from yeast to plants, is a Cu-binding site (Pufahl et al., 1997; Shin et al., 2012; Smith et al., 2017). A Blastp search showed that four residues—i.e., methionine, serine, and two cysteines in the MSCGGC motif of the putative CoIct1 amino acids sequence are predicted to form a heavy metal-binding site. To examine whether these amino acids in CoIct1 contribute to the ferimzone sensitivity and pathogenicity of *C. orbiculare*, we constructed mutant alleles of *CoICT1*, in which each M12, S13, C14, and C17 in the putative metal-binding site was replaced with alanine, and introduced them into the *coict1* mutant (Figure 4A). The generated transformants are hereafter referred to as CoICT1^M12A^, CoICT1^S13A^, CoICT1^C14A^, and CoICT1^C17A^. Ferimzone sensitivity on PDA medium containing ferimzone showed that the colony growth and colony color of CoICT1^M12A^, CoICT1^C14A^ and CoICT1^C17A^ were similar to those of the *coict1* mutant (Figure 4B). In contrast, the colony growth and colony color of CoICT1^S13A^ were similar to those of the wild-type and *CoICT1*-complemented transformant (Figures 4B and 4C). Next, these alanine-scanning transformants were tested for their pathogenicity using cucumber cotyledons. The CoICT1^M12A^, CoICT1^C14A^, and CoICT1^C17A^ transformants failed to develop lesions, whereas CoICT1^S13A^ developed lesions on cumber cotyledons that were similar to those induced by the wild-type and *CoICT1*-complemented transformant (Figure 4D). Moreover, the CoICT1^M12A^, CoICT1^C14A^, and CoICT1^C17A^ transformants formed appressoria with a pale brown color (Figures 4E and 4F). These results suggested that methionine and cysteine residues in the MSCGGC (12-17aa) sequence of CoIct1 are required for the ferimzone sensitivity and pathogenicity of *C. orbiculare*.

### *CoCCC2* Is Involved in Ferimzone Sensitivity and Pathogenicity

**Figure 5:**
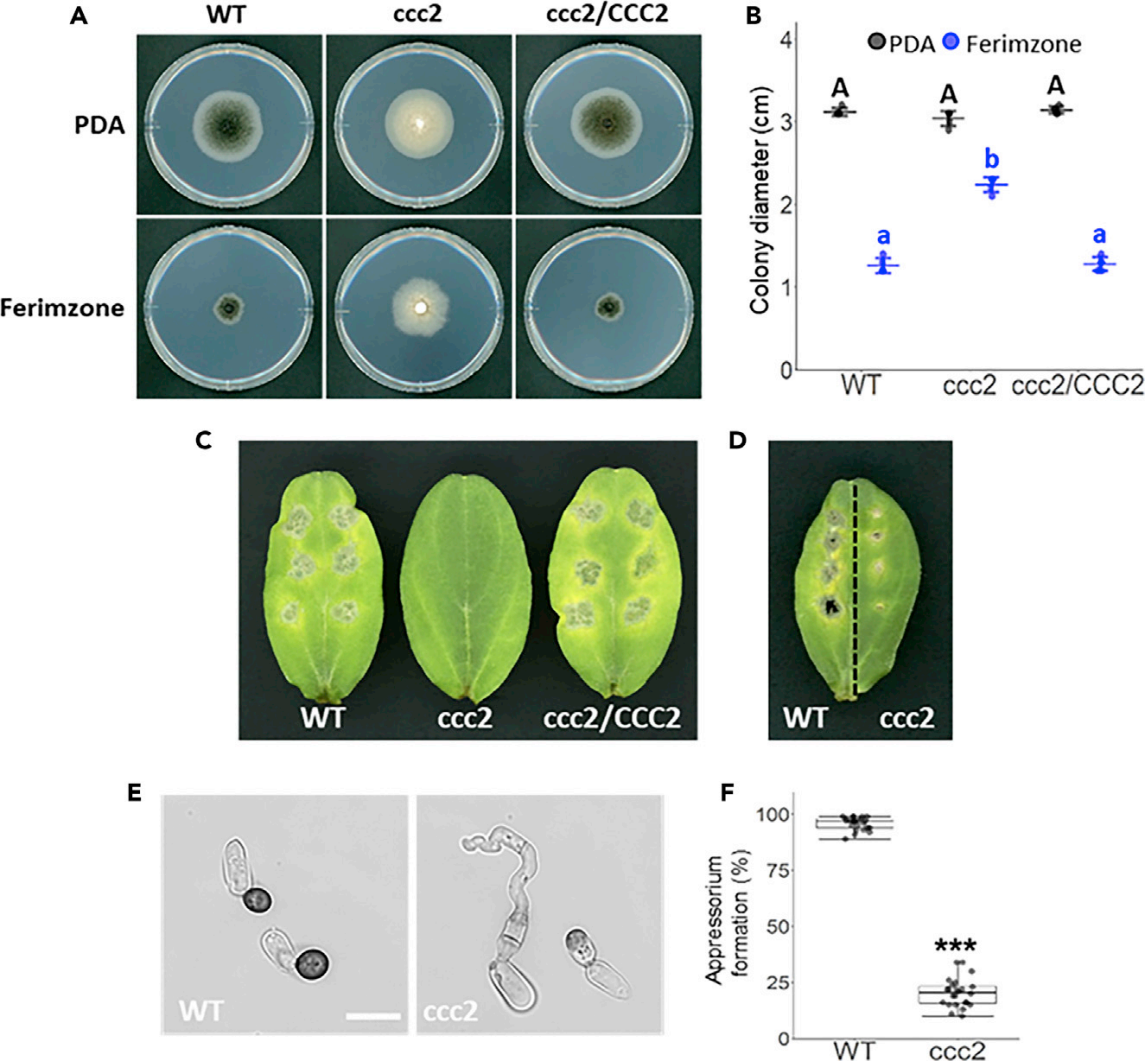
CoCcc2 Is Required for Ferimzone Sensitivity and Pathogenicity (A) Ferimzone-sensitivity tests in the *coccc2* mutant. A mycelia block of each strain was placed on ferimzone-containing PDA medium and was incubated for six days at 24°C. WT, wild-type; ccc2, *coccc2* mutant; ccc2/CCC2, *CoCCC2*-complemented transformant. (B) Average colony diameter in the *coccc2* mutant on the ferimzone-containing PDA medium. Error bars represent standard deviation of the mean (n = 5). Different letters above the scatter plots of each column represent significant differences (Tukey's HSD test; p < 0.001). (C) Pathogenicity assays on intact cucumber cotyledons. Conidial suspensions of each strain were dropped onto the adaxial surface of cucumber cotyledons and inoculated leaves were incubated for six days at 24°C. (D) Pathogenicity assays on wounded cucumber cotyledons. Conidial suspensions of each strain were dropped onto the wounded sites of cucumber cotyledons and inoculated leaves were incubated for six days at 24°C. (E) Appressorium formation in the *coccc2* mutant. Conidial suspensions of each strain were placed on cover slips and incubated for 24 h at 24°C. Scale bar, 10 μm. (F) Percentage of appressorium in the *coccc2* mutant. Approximately, 100 conidia were observed per one well. Eight wells were observed in one experiment and three independent experiments were conducted. Plotted dot represents appressorium formation (%) in each well. The asterisk represents a significant difference between the wild-type and the *coccc2* mutant (Mann-Whitney U t test: ∗∗∗p < 0.001).

In *S. cerevisia*e, Atx1, a homolog of CoIct1, transports Cu ions to the Golgi apparatus through P-type ATPase Ccc2 (Pufahl et al., 1997; Lin et al., 1997). We searched for a homolog gene of *S. cerevisiae CCC2* in *C. orbiculare* using a BLASTp search and found a *CoCCC2* gene which putatively encodes 1167 amino acids with the P-type ATPase motif and four HMA domains. The amino acid sequence deduced from *CoCCC2* shared 37% identity (E value = 0.0) to that of *S. cerevisiae* Ccc2. To examine the involvement of *CoCCC2* in ferimzone sensitivity, a *coccc2* mutant was generated by AtMT through double-crossover homologous recombination, and the disruption of the targeted gene was confirmed by Southern blot analysis (Figure S4B. Confirmation of Targeted Gene Disruptions by Southern Blotting Analyses, Related to Figure 5). On the PDA medium, a colony of the *coccc2* mutant showed a pale orange color (Figure 5A). The *coccc2* mutant formed a larger colony than the wild-type on the ferimzone-containing PDA medium (Figures 5A and 5B). The pathogenicity test using cucumber leaves showed that the *coccc2* mutant lacked the ability to cause disease symptoms on either intact or wounded leaves (Figures 5C and 5D). These phenotypes suggested that *CoCCC2* is required for ferimzone sensitivity and pathogenicity.

Microscopic observation showed that approximately 80% of conidia of the *coccc2* mutant failed to differentiate appressoria and the remainder formed pale brown-colored appressoria (Figures 5E and 5F). This result indicated that *CoCCC2* is required not only for melanization but also for appressorial differentiation.

### *MoICT1* and *MoCCC2* Are Involved in Ferimzone Sensitivity and Pathogenicity of *M. oryzae*

**Figure 6:**
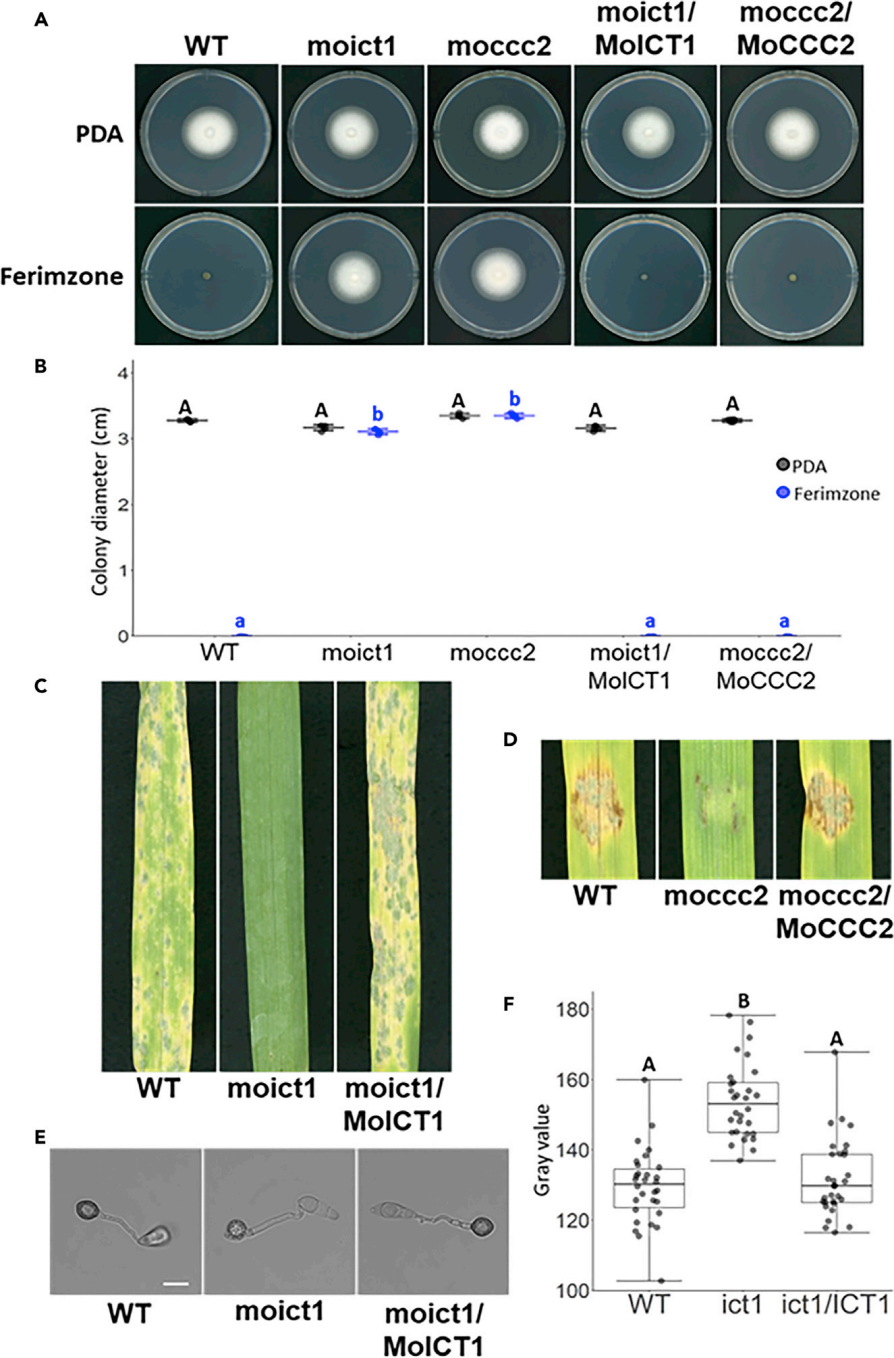
*ICT1* and *CCC2* Are Required for Ferimzone Sensitivity and Pathogenicity in *M. oryzae* (A) Ferimzone-sensitivity tests of the *moict1* mutant and the *moccc2* mutant. A mycelia block of each strain was placed on ferimzone-containing PDA medium and was incubated for six days at 24°C. WT, wild-type; moict1, *moict1* mutant; ict1/ICT1, *MoICT1*-complemented transformant; moccc2, *moccc2* mutant; moccc2/MoCCC2, MoCCC2-complemented transformant. (B) Average colony diameter in the *moict1* mutant the *moccc2* mutants on the ferimzone-containing PDA medium. Error bars represent standard deviation of the mean (n = 5). Different letters above the scatter plots of each column represent significant differences (Tukey's HSD test; p < 0.001). (C) Pathogenicity assays of the *moict1* mutant on barley leaves. Conidial suspensions of each strain were dropped onto the surface of barley leaves and inoculated leaves were incubated for six days at 24°C. (D) Pathogenicity assays of the *moccc2* mutant on barley leaves. Mycelial blocks of each strain were placed onto the surface of barley leaves and inoculated leaves were incubated for six days at 24°C. (E) Appressorial melanization of the *moict1* mutant. Conidial suspensions of each strain were placed on cover slips and incubated for 24 h at 24°C. Scale bar, 10 μm. (F) Gray scale value of appressorial melanization of the *moict1* mutant. Approximately, 30 appressoria of each strain were measured. Different letters above the box plots represent significant differences (Tukey's HSD test; p < 0.001).

Ferimzone is used for the control of rice diseases particularly rice blast disease caused by *M. oryzae*, which forms melanized appressoria like *C. orbiculare*. We searched for homologous genes of *CoICT1* and *CoCCC2* by BLASTp in *M. oryzae* and found *MoICT1* and *MoCCC2*. Amino acid sequences deduced from MoIct1 and MoCcc2, respectively, shared 98% and 74% identity to those from CoIct1 and CoCcc2 of *C. orbiculare*. To characterize *MoICT1* and *MoCCC2* in terms of ferimzone sensitivity and pathogenicity, we generated *moict1* and *moccc2* mutants by AtMT through double-crossover homologous recombination. The disruption of each targeted gene was confirmed by Southern blot analysis (Figures S4C and S4D). Confirmation of Targeted Gene Disruptions by Southern Blotting Analyses, Related to Figure 6). We tested *moict1* and *moccc2* mutants for their sensitivity to ferimzone on PDA containing ferimzone at the concentration of 2.5 μg/mL, in which mycelial growth of the wild-type *M. oryzae*, *MoICT1-*and *MoCCC2*-complemented transformants was almost completely inhibited (Figures 6A and 6B). On the ferimzone-containing PDA medium, both mutants formed colonies whose sizes were equivalent to those formed in the absence of ferimzone (Figures 6A and 6B).

Next, to examine whether *MoICT1* and *MoCCC2* are involved in pathogenicity, we performed inoculation assays of *moict1* and *moccc2* mutants using a barley cultivar (Nigrate) that is highly susceptible to *M. oryzae*. Mycelial blocks the *moccc2* mutant were used in the inoculation assay because this mutant is completely defective in conidiation. Both mutants failed to cause disease symptoms under the experimental conditions by which the wild-type and complementation transformants induced disease symptoms (Figures 6C and 6D). Moreover, microscopic observation revealed that the *moict1* mutant formed non-melanized appressoria (Figures 6E and 6F). These phenotypes of the *moict1* and *moccc2* mutants were similar to those of the *coict1* and *coccc2* mutants, suggesting that the molecular mechanisms underlying ferimzone sensitivity and pathogenicity were shared in common between *M. oryzae* and *C. orbiculare*.

## Discussion

We found that *CoICT1* is involved in ferimzone sensitivity by analyzing a mutant obtained by screening T-DNA-inserted *C. orbiculare*. Copper transporter Atx1, a homolog of Ict1, and Ccc2 are known to be involved in melanization in the human pathogenic fungus *C. neoformans* (Walton et al., 2005). The phenotypes of the *ict1* and *ccc2* mutants in *C. orbiculare* and *M. oryzae* showed the conservation of regulatory roles of the copper transport pathway in melanin biosynthesis in filamentous fungi. Microscopic observations of the mCherry:CoICT1-complemented transformant showed that CoIct1 was preferentially expressed and localized in melanized appressoria, consistent with the CoLac2 expression patterns (Lin et al., 2012). Moreover, the extracellular laccase activity was much weaker in the *coict1* mutant than in the wild-type. These results suggested that CoIct1 serves as a copper transporter to target cuproenzyme laccase. *CLAP1*, a homolog of *CCC2* in *S. cerevisiae*, also contributes to melanization via laccase activity in *C. lindemuthianum,* and the *coccc2* mutant formed non-melanized colony and appressoria (Parisot et al., 2002). Therefore, the copper transport pathway, consisting of CoIct1 and CoCcc2, appears to play a key role in the regulation of laccase. Although the *coict1* and *coccc2* mutants showed low sensitivity to ferimzone, melanin biosynthesis gene-disrupted mutants showed similar levels of ferimzone sensitivity as the wild-type. This result indicated that ferimzone does not directly target the melanin biosynthesis pathway. Interestingly, the colony color of the *coict1* mutant was clearly weaker in the incubation with ferimzone compared to the incubation without ferimzone (Figure 1A). The alanine-scanning transformants (CoICT1^M12A^, CoICT1^C14A^, CoICT1^C17A^) also showed phenotypes similar to that of the *coict1* mutant (Figure 4B). These results suggested that ferimzone induces the attenuation of melanization when CoIct1 is dysfunctional. We wondered whether both the ferimzone and CuSO_4_-treated *coict1* mutant could grow normally and form a normal colony with melanin pigmentation, whose phenotypes have a potential risk for the manifestation of ferimzone-resistant strains. Under this condition, the *coict1* mutant formed the colony pigmented with melanin, but the mutant was sensitive to ferimzone, suggesting that the threat to ferimzone resistance could be circumvented. Based on the experimental evidence, we concluded that there is a trade-off relation between ferimzone sensitivity and melanin biosynthesis, which is necessary for fungal pathogenicity through copper transport pathways.

Fungi must control copper status in order to maintain proper cell function and have therefore evolved networks to drive Cu uptake, efflux and sequestration in response to environmental conditions (Marvin et al., 2004., Chun and Madhani, 2010; Ding et al., 2013; Park et al., 2014; Wiemann et al., 2017). Previous studies have revealed that Atx1 delivers copper ions to Ccc2 on the Golgi apparatus, but its function as a regulator of homeostasis is not defined in *C. neoformans* (Walton et al., 2005). In this study, vegetative hyphae of the *coict1* mutant accumulated equivalent amounts of Cu as the wild-type, suggesting that CoIct1 is not a key regulator of copper homeostasis. Because the metal-binding site in CoIct1 is important for sensitivity to ferimzone (Figure 4), it is possible that ferimzone affects Cu homeostasis by disturbing CoIct1-mediated copper ion transport. However, ferimzone had no effect on intracellular Cu levels in the wild-type. Therefore, inhibition of hyphal growth by ferimzone seems not to be associated with excess Cu or Cu deficiency. In *Arabidopsis thaliana*, root length and growth are hypersensitive to excess Cu in an *atx1* mutant, and Atx1 expression increases in the presence of Cu (Shin et al., 2012). We showed that the level of melanization in a CuSO_4_-treated *coict1* mutant was restored to that in the wild-type, and the mutant exhibited normal colony growth. Moreover, CoIct1 was not increased in CuSO_4_-treated vegetative hyphae of the wild-type (Figure 3F). These results suggested that CoIct1 appears not to be engaged in the control of copper homeostasis in response to high extracellular Cu levels, unlike in the case of *A. thaliana* Atx1.

Interestingly, we observed that the combined presence of ferimzone and CuSO_4_ enhanced the accumulation of Cu in the *coict1* mutant, but not in the wild-type (Figure 3E). It should be noted that the amount of Cu in the *coict1* mutant was similar to that in the wild-type either in the absence or presence of ferimzone or CuSO_4_ (Figure 3E). This raises a question. How does the combination of ferimzone and CuSO_4_ trigger aberrant Cu accumulation specifically in the *coict1* mutant? A previous study revealed that ferimzone disturbs the uptake of acetic acid and facilitates the leakage of acidic electrolytes from the vegetative hyphae of *Pyricularia oryzae* (Okuno et al., 1989b). A plausible hypothesis is that, upon loss of CoIct1 functions, ferimzone might facilitate Cu uptake from exogenously supplied CuSO_4_ and/or suppress Cu efflux, which would subsequently lead to aberrant Cu accumulation to cytotoxic levels. Our findings suggest that the combined use of ferimzone and Cu could suppress and circumvent the occurrence of ferimzone-resistant strains of fungi.

The chemical genetics approach is a powerful tool for unraveling the hidden functions of genes. In *A. thaliana*, screening using the small molecule Triplin, a copper iron chelator, revealed that Atx1 transports copper irons to Cu-transporting P-type ATPase Ran1, which plays a role in copper transport to the ethylene sensor Etr1 (Rodríguez et al., 1999; Li et al., 2017). In this study, forward genetic screening using ferimzone revealed that CoIct1 is involved in hyphal growth of *C. orbiculare*. However, the molecular mechanisms underlying CoIct1-mediated regulation of hyphal growth remain unknown. The phenotypes of alanine-scanning mutants of *CoICT1* implied that heavy metal ions including copper ions would be key factors for the regulation of hyphal growth. Cu/Zn superoxide dismutases (SODs), which convert O_2_^-^ into H_2_O_2,_ serve to control ROS, which functions as signaling molecules for the induction of secondary metabolism and morphogenesis in fungi (Narasipura et al., 2003; Ding et al., 2014). A copper-bound form of the copper chaperone Ccs1 interacts with an inactivated form of SODs and delivers the copper necessary for their activities in *S. cerevisiae* (Rae et al., 1999; Furukawa et al., 2004). *MoNOX1* and *MoNOX2*, members of the superoxide-generating NADPH oxidase family, regulate hyphal growth and trigger ROS accumulations in hyphal tips in *M. oryzae* (Egan et al., 2007). Therefore, CoIct1-mediated copper transport may regulate ROS generations which drive the hyphal growth. Further studies are needed to elucidate the relations between CoIct1 and ROS generation.

### Limitations of the Study

In this study, we revealed that there is a trade-off relation between the sensitivity to the fungicide ferimzone and the copper transport-mediated melanin biosynthesis necessary for fungal infection. Although the metal-binding motif is a crucial site of the ferimzone sensitivity, we raise a question whether ferimzone binds to this site directly or affects the metal-binding indirectly. Approaches of the crystal analysis of Ict1 and protein-chemical interactions would unravel the mode of action of ferimzone. As described in the discussion section, further research is needed to elucidate a relation between copper transporters and the regulation of hyphal growth of fungi.

### Resource Availability

#### Lead Contact

Further information and requests for resources and reagents should be directed to and will be fulfilled by the Lead Contact, Ken Harata (a16029@mail.ryukoku.ac.jp).

#### Materials Availability

This study did not generate new unique reagents.

#### Data and Code Availability

We did not use any data sets.

## Methods

All methods can be found in the accompanying Transparent Methods supplemental file.

## Supplemental Information

Document S1. Transparent Methods, Figures S1–S7, and Table S1

## References

[bib1] Castroagudín V.L., Ceresini P.C., de Oliveira S.C., Reges J.T., Maciel J.L., Bonato A.L., Dorigan A.F., McDonald B.A. (2015). Resistance to QoI fungicides is widespread in Brazilian populations of the wheat blast pathogen *Magnaporthe oryzae*. Phytopathology.

[bib2] Chun C.D., Madhani H.D. (2010). Ctr2 links copper homeostasis to polysaccharide capsule formation and phagocytosis inhibition in the human fungal pathogen *Cryptococcus neoformans*. PLoS One.

[bib3] Dean R., Van Kan J.A., Pretorius Z.A., Hammond-Kosack K.E., Di Pietro A., Spanu P.D., Rudd J.J., Dickman M., Kahmann R., Ellis J. (2012). The Top 10 fungal pathogens in molecular plant pathology. Mol. Plant Pathol..

[bib4] Ding C., Festa R.A., Chen Y.L., Espart A., Palacios Ò., Espín J., Capdevila M., Atrian S., Heitman J., Thiele D.J. (2013). *Cryptococcus neoformans* copper detoxification machinery is critical for fungal virulence. Cell Host Microbe.

[bib5] Ding C., Festa R.A., Sun T.S., Wang Z.Y. (2014). Iron and copper as virulence modulators in human fungal pathogens. Mol. Microbiol..

[bib6] Egan M.J., Wang Z.Y., Jones M.A., Smirnoff N., Talbot N.J. (2007). Generation of reactive oxygen species by fungal NADPH oxidases is required for rice blast disease. Proc. Natl. Acad. Sci. U S A.

[bib7] Furukawa Y., Torres A.S., O’Halloran T.V. (2004). Oxygen-induced maturation of SOD1: a key role for disulfide formation by the copper chaperone CCS. EMBO J..

[bib8] Harata K., Okuno T. (2019). Threonine synthase *CoTHR4* is involved in infection-related morphogenesis during the pre-penetration stage in *Colletotrichum orbiculare*. Microb. Pathog..

[bib9] Inoue Y., Vy T.T.P., Yoshida K., Asano H., Mitsuoka C., Asuke S., Anh V.L., Cumagun C.J.R., Chuma I., Terauchi R. (2017). Evolution of the wheat blast fungus through functional losses in a host specificity determinant. Science.

[bib10] Islam M.T., Croll D., Gladieux P., Soanes D.M., Persoons A., Bhattacharjee P., Hossain M.S., Gupta D.R., Rahman M.M., Mahboob M.G. (2016). Emergence of wheat blast in Bangladesh was caused by a South American lineage of *Magnaporthe oryzae*. BMC Biol..

[bib11] Khush G.S. (2005). What it will take to feed 5.0 billion rice consumers in 2030. Plant Mol. Biol..

[bib12] Kubo Y., Takano Y. (2013). Dynamics of infection-related morphogenesis and pathogenesis in *Colletotrichum orbiculare*. J. Gen. Plant Pathol..

[bib13] Kubo Y., Nakamura H., Kobayashi K., Okuno T., Furusawa I. (1991). Cloning of a melanin biosynthetic gene essential for appressorial penetration of *Colletotrichum lagenarium*. Mol. Plant Microbe Interact..

[bib14] Kubo Y., Takano Y., Endo N., Yasuda N., Tajima S., Furusawa I. (1996). Cloning and structural analysis of the melanin biosynthesis gene *SCD1* encoding scytalone dehydratase in *Colletotrichum lagenarium*. Appl. Environ. Microbiol..

[bib15] Li W., Lacey R.L., Ye Y., Lu J., Yeh K.C., Xiao Y., Li L., Wen C.K., Binder B.M., Zhao Y. (2017). Triplin, a small molecule, reveals copper ion transport in ethylene signaling from ATX1 to RAN1. PLoS Genet..

[bib16] Lin S.J., Pufahl R.A., Dancis A., O'Halloran T.V., Culotta V.C. (1997). A role for the *Saccharomyces cerevisiae ATX1* gene in copper trafficking and iron transport. J. Biol. Chem..

[bib17] Lin S.Y., Okuda S., Ikeda K., Okuno T., Takano Y. (2012). *LAC2* encoding a secreted laccase is involved in appressorial melanization and conidial pigmentation in *Colletotrichum orbiculare*. Mol. Plant Microbe Interact..

[bib18] Marvin M.E., Mason R.P., Cashmore A.M. (2004). The *CaCTR1* gene is required for high-affinity iron uptake and is transcriptionally controlled by a copper-sensing transactivator encoded by *CaMAC1*. Microbiology.

[bib19] Narasipura S.D., Ault J.G., Behr M.J., Chaturvedi V., Chaturvedi S. (2003). Characterization of Cu,Zn superoxide dismutase (SOD1) gene knock-out mutant of *Cryptococcus neoformans* var. gattii: role in biology and virulence. Mol. Microbiol..

[bib20] Okuno T., Furusawa I., Matsuura K., Shishiyama J. (1989). Mode of action of ferimzone, a novel systematic fungicide for rice disease: biological properties against *Pyricularia oryzae* in vitro. Phytopathology.

[bib21] Okuno T., Furusawa I., Matsuura K., Shishiyama J. (1989). Mode of action ferimzone (TF-164), a novel systematic fungicide for rice disease: effects on the general metabolism of *Pyricularia oryzae*. Ann. Phytopathol Soc. Jpn..

[bib22] Parisot D., Dufresne M., Veneault C., Laugé R., Langin T. (2002). clap1, a gene encoding a copper-transporting ATPase involved in the process of infection by the phytopathogenic fungus *Colletotrichum lindemuthianum*. Mol. Genet. Genomics.

[bib23] Park Y.S., Lian H., Chang M., Kang C.M., Yun C.W. (2014). Identification of high-affinity copper transporters in *Aspergillus fumigatus*. Fungal Genet. Biol..

[bib24] Perpetua N.S., Kubo Y., Yasuda N., Takano Y., Furusawa I. (1996). Cloning and characterization of a melanin biosynthetic *THR1* reductase gene essential for appressorial penetration of *Colletotrichum lagenarium*. Mol. Plant Microbe Interact..

[bib25] Pufahl R.A., Singer C.P., Peariso K.L., Lin S.J., Schmidt P.J., Fahrni C.J., Culotta V.C., Penner-Hahn J.E., O'Halloran T.V. (1997). Metal ion chaperone function of the soluble Cu(I) receptor Atx1. Science.

[bib26] Rae T.D., Schmidt P.J., Pufahl R.A., Culotta V.C., O'Halloran T.V. (1999). Undetectable intracellular free copper: the requirement of a copper chaperone for superoxide dismutase. Science.

[bib27] Rodríguez F.I., Esch J.J., Hall A.E., Binder B.M., Schaller G.E., Bleecker A.B. (1999). A copper cofactor for the ethylene receptor *ETR1* from Arabidopsis. Science.

[bib28] Saitoh Y., Izumitsu K., Morita A., Tanaka C. (2010). A copper-transporting ATPase *BcCCC2* is necessary for pathogenicity of *Botrytis cinerea*. Mol. Genet. Genomics.

[bib29] Shin L.J., Lo J.C., Yeh K.C. (2012). Copper chaperone antioxidant protein1 is essential for copper homeostasis. Plant Physiol..

[bib30] Smith A.D., Logeman B.L., Thiele D.J. (2017). Copper acquisition and utilization in fungi. Annu. Rev. Microbiol..

[bib31] Takagaki M., Kaku K., Watanabe S., Kawai K., Shimizu T., Sawada H., Kumakura K., Nagayama K. (2004). Mechanism of resistance to carpropamid in *Magnaporthe grisea*. Pest Manag. Sci..

[bib32] Takano Y., Kubo Y., Shimizu K., Mise K., Okuno T., Furusawa I. (1995). Structural analysis of *PKS1*, a polyketide synthase gene involved in melanin biosynthesis in *Colletotrichum lagenarium*. Mol. Gen. Genet..

[bib33] Tsuji G., Fujii S., Fujihara N., Hirose C., Tsuge S., Shiraishi T., Kubo Y. (2003). *Agrobacterium tumefaciens*-mediated transformation for random insertional mutagenesis in *Colletotrichum lagenarium*. J. Gen. Plant Pathol..

[bib34] Walton F.J., Idnurm A., Heitman J. (2005). Novel gene functions required for melanization of the human pathogen *Cryptococcus neoformans*. Mol. Microbiol..

[bib35] Wiemann P., Perevitsky A., Lim F.Y., Shadkchan Y., Knox B.P., Figueora J.A.L., Choera T., Niu M., Steinberger A.J., Wüthrich M. (2017). *Aspergillus fumigatus* copper export machinery and reactive oxygen intermediate defense counter host copper-mediated oxidative antimicrobial offense. Cell Rep..

