# Supplementary material for: Trade-Off Relation between Fungicide Sensitivity and Melanin Biosynthesis in Plant Pathogenic Fungi
Source: iScience. 2020 Oct 8;23(11):101660. doi: 10.1016/j.isci.2020.101660 (PMC7582099; doi:10.1016/j.isci.2020.101660)
Supplement: Document S1. Transparent Methods, Figures S1–S7, and Table S1 [file mmc1.pdf]

iScience, Volume *23*

Supplemental Information

Trade-Off Relation between Fungicide Sensitivity and Melanin Biosynthesis in Plant Pathogenic Fungi

Ken Harata, Hiroyuki Daimon, and Tetsuro Okuno

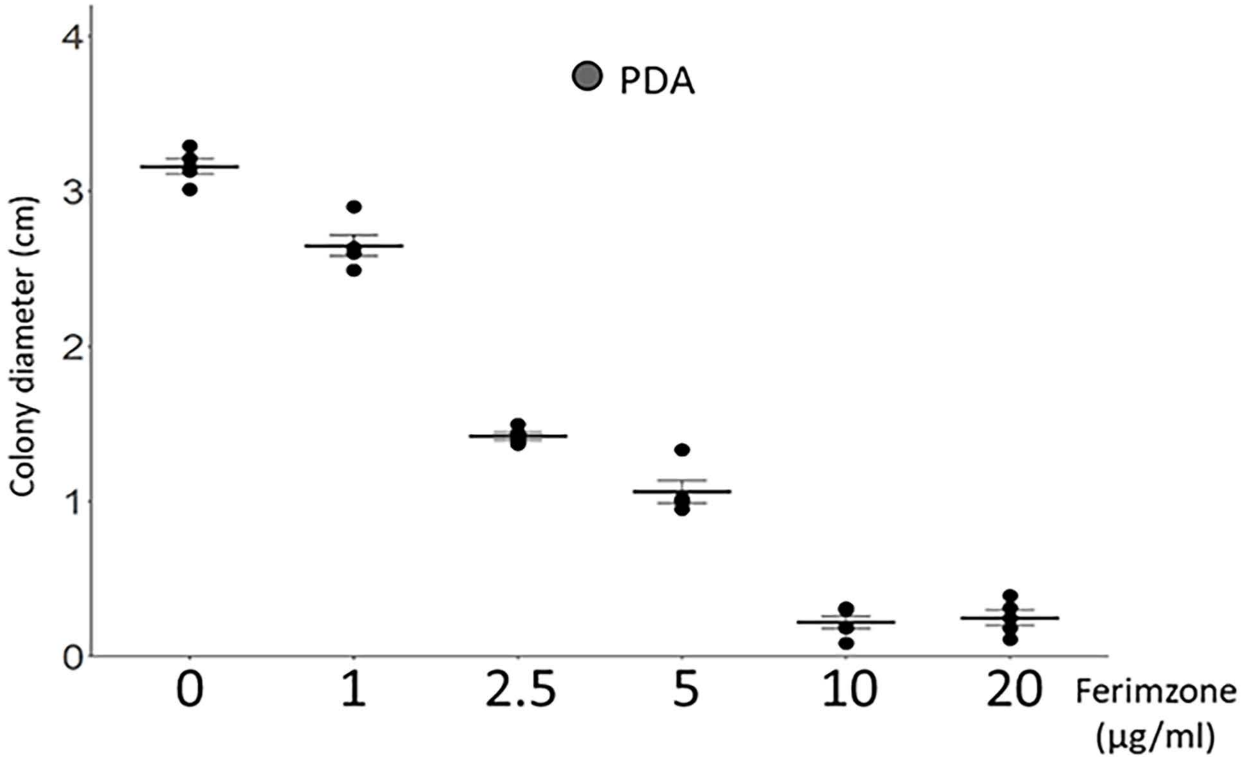

Figure S1. Average Colony Diameter in the Wild-type on the PDA Medium Containing Different Concentrations of Ferimzone. Related to Figure 1 and 6

Ferimzone-sensitivity tests of the wild-type. A mycelial block of the wild-type was placed on ferimzone-containing PDA medium and was incubated for 6 days at 24℃. Error bars represent standard deviation of the mean (n = 5). 0; PDA medium, 1; 1 μg/ml ferimzone-containing PDA medium, 2.5; 2.5 μg/ml ferimzone-containing PDA medium, 5; 5 μg/ml ferimzone-containing PDA medium, 10; 10 μg/ml ferimzone-containing PDA medium, 20; 20 μg/ml ferimzone-containing PDA medium.

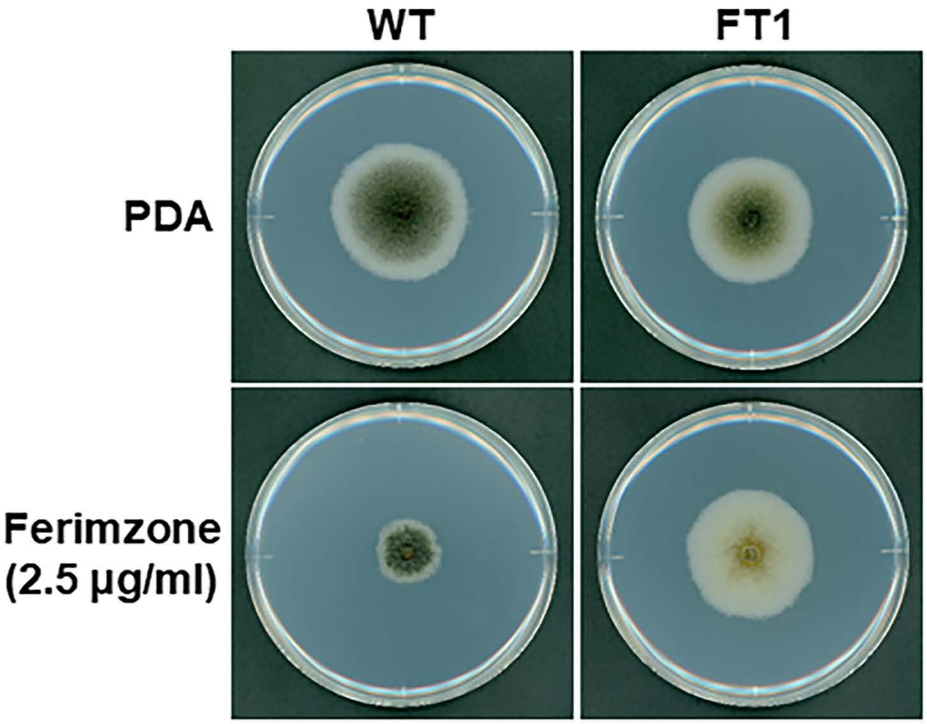

Figure S2. The FT1 Transformant Shows a Low Sensitivity to Ferimzone. Related to Figure 1

A ferimzone-tolerance assay of the FT1 transformant. A mycelial block of each strain was placed on PDA medium containing 2.5 µg/ml ferimzone and was incubated for 6 days at 24℃. WT, wild-type; FT1, the T-DNA insertion transformant with low ferimzone-sensitivity.

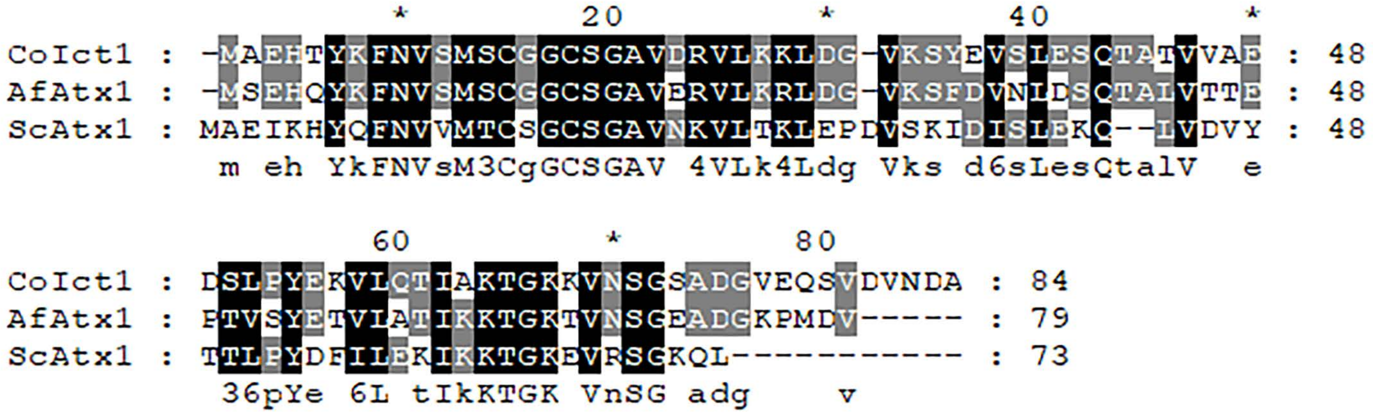

Figure S3. The Amino Acid Sequence of Colct1 Shows High Homology with Atx1 of *A. fumigatus* and *S. cerevisiae*. Related to Figure 1

The amino acids of *C. orbiculare* Colct1 were aligned with those of *A. fumigatus* Atx1 and *S. cerevisiae* Atx1 using the Clustal W program. Numbers on the right indicate amino acid residue positions. Identical amino acids are indicated by a black background, similar residues are indicated by a gray background and gaps introduced for alignments are indicated by a hyphen using GeneDoc.

Figure S4

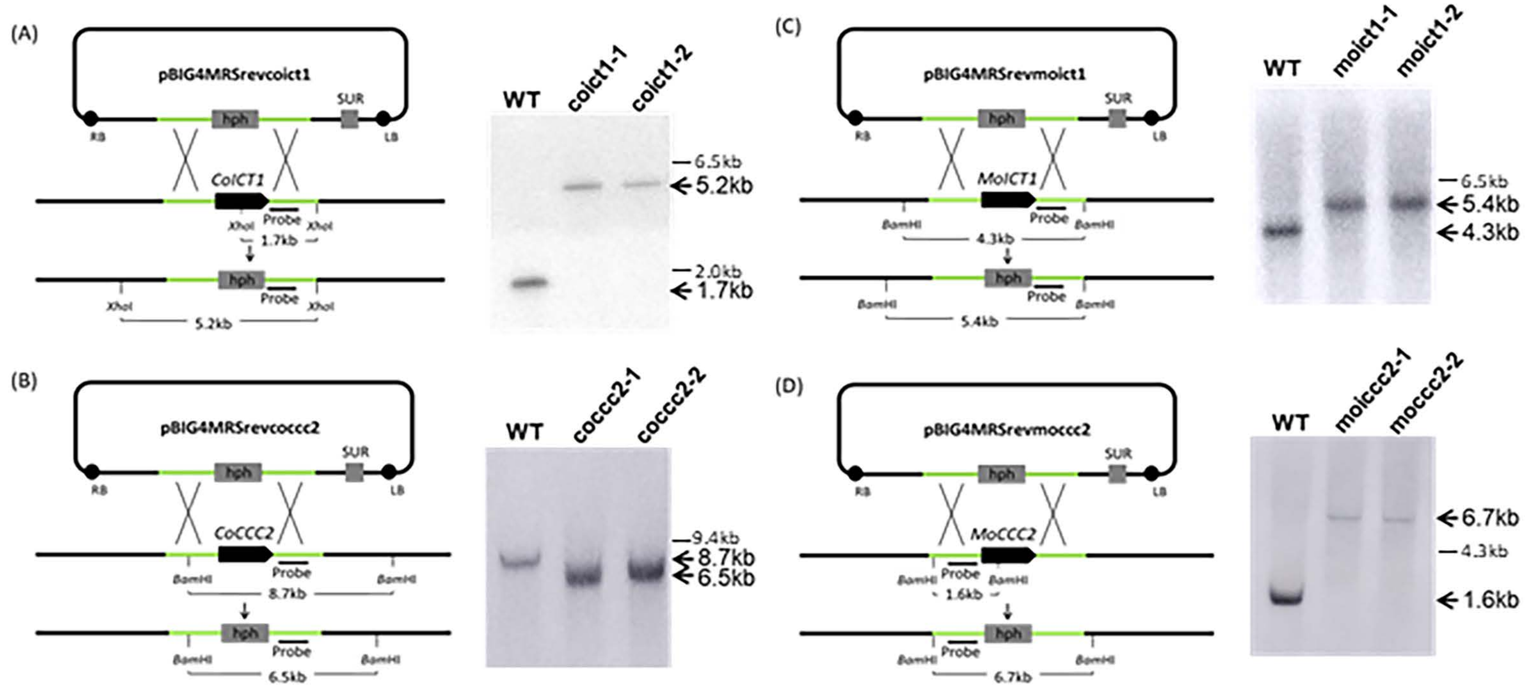

Figure S4. Confirmation of Targeted Gene Disruptions by Southern Blotting Analyses. Related to Figure 1, 5 and 6

(A) A schematic diagram of the *CoICT1* gene disruption construct in *C. orbiculare* by *Agrobacterium tumefaciens*-mediated transformation with the *coict1* disruption vector to replace a hygromycin phosphotransferase gene (hph) fragment with the *CoICT1* gene. Bars represent probes for DNA gel blot. Following double-crossover homologous recombination, an *Xho*I fragment of approximately 1.7 kb containing *CoICT1* in the wild-type is predicted to be replaced by a fragment of approximately 5.2 kb containing the hph fragment. *CoICT1* gene disruption was confirmed by Southern blot analysis. Genomic DNAs from the wild-type 104-T and transformants were digested with *Xho*I and the probe with an upstream 1.0 kb fragment of the *CoICT1* gene. **(B)** A schematic diagram of the *CoCCC2* gene disruption construct in *C. orbiculare* by *Agrobacterium tumefaciens*-mediated transformation with the *coccc2* disruption vector to replace the hygromycin phosphotransferase gene (hph) fragment with the *CoCCC2* gene. Bars represent probes for the DNA gel blot. Following double-crossover homologous recombination, a *Bam*HI fragment of approximately 8.7 kb containing *CoCCC2* in the wild-type is predicted to be replaced by a fragment of approximately 6.5 kb containing the hph fragment. *CoCCC2* gene disruption was confirmed by Southern blot analysis. Genomic DNAs from the wild-type 104-T and transformants were digested with *Bam*HI and probed with a downstream 1.0 kb fragment of the *CoCCC2* gene. **(C)** A schematic diagram of the *MoICT1* gene disruption construct in *M. oryzae* by *Agrobacterium tumefaciens*-mediated transformation with the *moict1* disruption vector to replace a hygromycin phosphotransferase gene (hph) fragment with the *MoICT1* gene. Bars represent probes for the DNA gel blot. Following double-crossover homologous recombination, a *Bam*HI fragment of approximately 4.3 kb containing *MoICT1* in the wild-type is predicted to be replaced by a fragment of approximately 5.4 kb containing the hph fragment. *MoICT1* gene disruption was confirmed by Southern blot analysis. Genomic DNAs from the wild-type 104-T and transformants were digested with *Bam*HI and probed with an downstream 1.0 kb fragment of the *MoICT1* gene. **(D)** A schematic diagram of the *MoCCC2* gene disruption construct in *M. oryzae* by *Agrobacterium tumefaciens*-mediated transformation with the *moccc2* disruption vector to replace a hygromycin phosphotransferase gene (hph) fragment with the *MoCCC2* gene. Bars represent probes for the DNA gel blot. Following double-crossover homologous recombination, a *Bam*HI fragment of approximately 1.6 kb containing *MoCCC2* in the wild-type is predicted to be replaced by a fragment of approximately 6.7 kb containing the hph fragment. *MoCCC2* gene disruption was confirmed by Southern blot analysis. Genomic DNAs from the wild-type 104-T and transformants were digested with *Bam*HI and probed with an upstream 1.0 kb fragment of the *MoCCC2* gene .

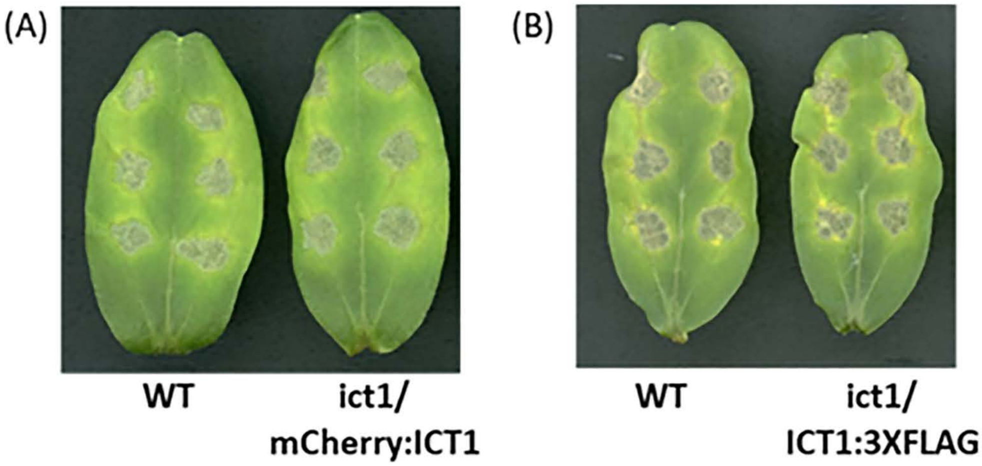

Figure S5. Pathogenicity Assays on mCherry:ICT1-Introduced Transformant and ICT1:3XFLAG-Introduced Transformant. Related to Figure 2 and 3

(A-B) Inoculation assays of the mCherry:ICT1-introduced transformant and ICT1:3XFLAG-introduced transformant on the intact leaves. Conidial suspensions (5.0×10^5 conidia/ml) of each strain were inoculated with the cucumber cotyledons and inoculated leaves were incubated for 6 days at 24℃. WT, wild-type; ict1/mCherry:ICT1, *ict1* mutant expressing the *mCherry:ICT1* fusion gene; Ict1/ICT1:3XFLAG, *ict1* mutant expressing the *ICT1*:3X*FLAG* fusion gene.

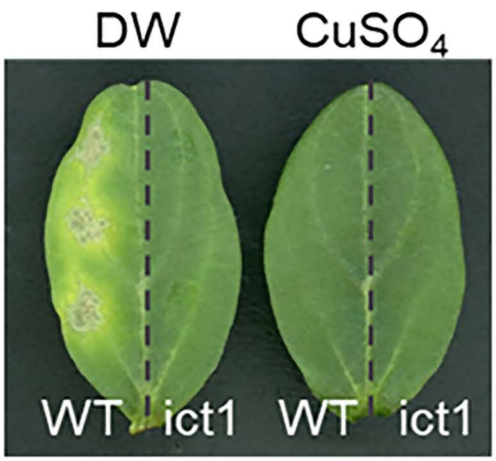

Figure S6. Pathogenicity Assays on $CuSO_4$-treated wild-type and *coict1* mutant. Related to Figure 2

Inoculation assays of $CuSO_4$-treated wild-type and *coict1* mutant on the intact leaves. Ten μM $CuSO_4$-treated conidial suspensions (5.0×10^5 conidia/ml) of each strain were inoculated with the cucumber cotyledons and inoculated leaves were incubated for 6 days at 24℃. WT, wild-type; ict1, *ict1* mutant.

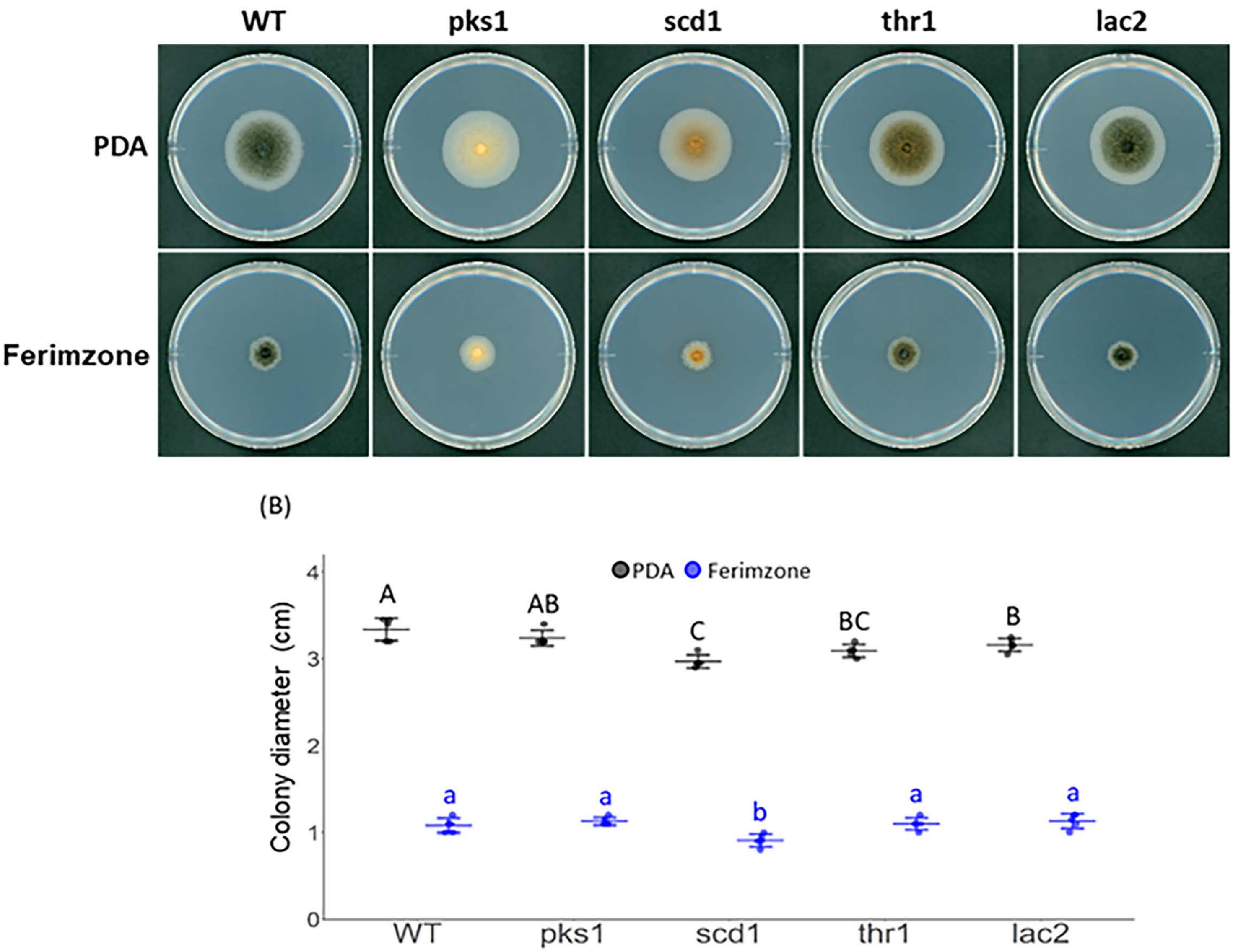

Figure S7. Ferimzone-Sensitivity Tests in Mutants of Melanin Biosynthesis Genes. Related to Figure 2

(**A**) The Colony in the melanin-deficient mutants on the ferimzone-supplemented PDA medium. Mycelia block of each strain was placed on ferimzone-supplemented PDA medium and was incubated for 6 days. WT; wild-type, pks1; *copks1* mutant, scd1; *coscd1* mutant, thr1; *cothr1* mutant, lac2; *colac2* mutant.

(**B**) Average of colony size in the melanin-deficient mutants on the ferimzone-supplemented PDA medium. Error bars represent standard deviation of the mean (n = 5). Different letters above scatter plots of each column represent significant differences (Tukey's HSD test; P < 0.01).

Table S1. PCR Primers Used in This Study. Related to Figure 1-6

Primer name	Sequence (5´→3´)	Subject
CoICT1F1B	TCCCTTAATTCTCCGTCTACAGCCTCAACCCCAAC	*CoICT1* gene complementation construct
CoICT1R1A	CAATCTGATCATGAGTAGATGCGCCTGCTCCTATT	
pBIG4MRBSF1A	CTCATGATCAGATTGTCGTTTCCCGCCTTCAGTTT	
pBIG4MRBSR1B	CGGAGAATTAAGGGAGTCACGTTATGACCTCTAGT	
HPHF1B	TGATATTGAAGGAGCATTTTTTGGGCTTGGCTGGA	*coict1* gene replacement construct
HPHR1A	TGGTTCCCGGTCGGCATCTACTCTATTCCTTTGCC	
pBIcoict1F1A	GCCGACCGGGAACCAGGTCTTGCGAGGAGTTTTTG	
pBIcoict1R1B	GCTCCTTCAATATCAACGAGGGAGTGTTTCTGTCG	
glymCherryF1A	GGTGGTGGTGGTGGTATGGTGAGCAAGGGCGAGGA	*CoICT1-mCherry* fusion gene construct
mCherryR1B	TTAAGATCTGTACAGCTCGTCCATGCCGCCGGTGG	
pBICoICT1mCF1A	CTGTACAGATCTTAAAAGCAGGACGTGATTGATTT	
pBICoICT1mCR1B	ACCACCACCACCACCTGCGTCGTTGACGTCGACGC	
CoICT1M12AF1B	GCGAGCTGCGGCGGCTGCTCCGGCGCCGTCGATCG	*CoICT1*M12A alanin scanning construct
CoICT1AlaR1A	AATCACGTCCTGCTTCTATGCGTCGTTGACGTCGA	
pBICoICT1AlaF1A	AAGCAGGACGTGATTGATTTCTTCTGGAAACGGGG	
pBICoICT1M12AR1B	GCCGCCGCAGCTCGCGCTGACGTTGAACTTGTAGG	
CoICT1S13AF1B	ATGGCCTGCGGCGGCTGCTCCGGCGCCGTCGATCG	*CoICT1*S13A alanin scanning construct
CoICT1AlaR1A	AATCACGTCCTGCTTCTATGCGTCGTTGACGTCGA	
pBICoICT1AlaF1A	AAGCAGGACGTGATTGATTTCTTCTGGAAACGGGG	
pBICoICT1S13AR1B	GCCGCCGCAGGCCATGCTGACGTTGAACTTGTAGG	
CoICT1C14AF1B	ATGAGCGCCGGCGGCTGCTCCGGCGCCGTCGATCG	*CoICT1*C14A alanin scanning construct
CoICT1AlaR1A	AATCACGTCCTGCTTCTATGCGTCGTTGACGTCGA	
pBICoICT1AlaF1A	AAGCAGGACGTGATTGATTTCTTCTGGAAACGGGG	
pBICoICT1C14AR1B	GCCGCCGGCGCTCATGCTGACGTTGAACTTGTAGG	
CoICT1C17AF2B	GCCTCCGGCGCCGTCGATCGCGTCCTCAAGAAGCT	*CoICT1*C17A alanin scanning construct
CoICT1AlaR1A	AATCACGTCCTGCTTCTATGCGTCGTTGACGTCGA	
pBICoICT1AlaF1A	AAGCAGGACGTGATTGATTTCTTCTGGAAACGGGG	
pBICoICT1C17AR2B	GACGGCGCCGGAGGCGCCGCCGCAGCTCATGCTGA	
CoICT1FLAGF1A	TCCACCCCAACAACAGGCGTCAAGTCTTACGAAGT	*CoICT1*-3X*FLAG* construct
CoICT1FLAGR1B	ACCCGCACCGCAGCCCGCTCGTTTCAAGGGTTATC	
pBISCoICT1glyFLAGF1B	GGCTGCGGTGCGGGTGCTGAATGTGCTTGCTGGGT	
pBISCoICTglyFLAGR1A	TGTTGTTGGGGTGGAAAAAGGATGTCAGCGCACAG	
CoCCC2F1B	TCCCTTAATTCTCCGATCAACAGGCCGATAAGGTG	*CoCCC2* gene complementation construct

CoCCC2R1A	CAATCTGATCATGAGACGACATGATCACGCTTCTG	
pBIG4MRBSF1A	CTCATGATCAGATTGTCGTTTCCCGCCTTCAGTTT	
pBIG4MRBSR1B	CGGAGAATTAAGGGAGTCACGTTATGACCTCTAGT	
HPHF1B	TGATATTGAAGGAGCATTTTTTGGGCTTGGCTGGA	*coccc2* gene replacement construct
HPHR1A	TGGTTCCCGGTCGGCATCTACTCTATTCCTTTGCC	
pBIcoccc2F1A	GCCGACCGGGAACCAATTCATGGAATCAGTATTCC	
pBIcoccc2R1B	GCTCCTTCAATATCATTGCCGCGTATACGGCTGAG	
CCC23HAF1A	ACGCGCCCGAGTTACATGACTGACCCGTCGACGCA	*CoCCC2*-3X*HA* construct
CCC23HAR1B	CGAAACCACAACAAGCTTGCAAGATGGGCTGATAA	
pBISCCC23HAF1B	CTTGTTGTGGTTTCGCCTAGCATAGCATCGGAGTC	
pBISCCC23HAR1A	GTAACTCGGGCGCGTCCAGAACTTCAGCAGCAAAC	
MoICT1F1B	TCCCTTAATTCTCCGCCTTGTGAACCATGGGCTAT	*MoICT1* gene complementation construct
MoICT1R1A	CAATCTGATCATGAGTTTTGACTCCTTTCGGGTTG	
pBIG4MRBSF1A	CTCATGATCAGATTGTCGTTTCCCGCCTTCAGTTT	
pBIG4MRBSR1B	CGGAGAATTAAGGGAGTCACGTTATGACCTCTAGT	
HPHF1B	TGATATTGAAGGAGCATTTTTTGGGCTTGGCTGGA	*moict1* gene replacement construct
HPHR1A	TGGTTCCCGGTCGGCATCTACTCTATTCCTTTGCC	
pBImoictF1A	GCCGACCGGGAACCAAAAACCCCGCTTTTGGGTCG	
pBImoictR1B	GCTCCTTCAATATCATTTGATTTGTTTGAAATGGG	
MoCCC2F1B	TCCCTTAATTCTCCGATTTCGCAAGTTTGGTTGCT	*MoCCC2* gene complementation construct
MoCCC2R1A	CAATCTGATCATGAGGAAGTTGTCCCTGCGAAAAG	
pBIG4MRBSF1A	CTCATGATCAGATTGTCGTTTCCCGCCTTCAGTTT	
pBIG4MRBSR1B	CGGAGAATTAAGGGAGTCACGTTATGACCTCTAGT	
HPHF1B	TGATATTGAAGGAGCATTTTTTGGGCTTGGCTGGA	*moccc2* gene replacement construct
HPHR1A	TGGTTCCCGGTCGGCATCTACTCTATTCCTTTGCC	
pBImoccc2F1A	GCCGACCGGGAACCACATGGACTGAGGTGCACCGG	
pBImoccc2R1B	GCTCCTTCAATATCACCGTGGTCAAATGGGGCGAC	

Transparent Methods

Fungal strains and fungal transformation

The *Colletotrichum orbiculare* strain 104-T and *Magnaporthe oryzae* strain P2 were used as the wild-types. All *C. orbiculare* and *M. oryzae* strains were cultured on PDA media (3.9% [w/v] PDA; Nissui Pharma) at 24 °C. For induction of conidia formation, *M. oryzae* strains were cultured on OTA media (7.25% [w/v] OTA; Difco Laboratories) under blue black light irradiation at 24 °C. *Agrobacterium tumefaciens*-mediated transformation (AtMT) and confirmation of targeted gene disruption by southern blot analysis were done as previously described (Tsuji et al., 2003; Harata and Kubo, 2014).

Screening of low sensitivity to ferimzone and identification of mutated genes

For screening of ferimzone-tolerant mutants, T-DNA insertional transformants generated by AtMT were incubated on PDA containing 10 μg/ml of ferimzone. After incubation for five days at 24 °C, we selected transformants that showed greater hyphal growth on ferimzone-containing media than the wild-type. Genomic DNA fragments flanking the inserted T-DNA in selected mutants were amplified by thermal asymmetrical interlaced PCR (Tail PCR) with specific primers and sequenced (Tsuji et al., 2003).

Plasmid construction for targeted gene disruption

All cloning for plasmid construction was carried out using an In-Fusion HD Cloning Kit (Clontech).

All primers used in this study are listed in Table S1. (Table S1. PCR Primers Used in This Study, Related to Figure 1-6).

For the construction of the *CoICT1* gene complementation plasmid (pBIG4MRSCoICT1), an approximately 4.2 kb *CoICT1* fragment and pBIG4MRSrev vector fragment, including the sulfonylurea-resistance gene, were amplified by PCR with appropriate primer pairs, respectively. pBIG4MRSCoICT1 was generated by insertion of the amplified *CoICT1* fragment into the linearized pBIG4MRSrev vector. *CoCCC2*, *MoICT1* and *MoCCC2* gene complementation plasmids were generated by the procedure described above.

For the construction of the *coict1* gene replacement plasmid (pBIG4MRScoict1), an approximately 1.4 kb hygromycin-resistance gene fragment (HPH) and a pBIG4MRSCoICT1 vector fragment not including the *CoICT1* ORF region were amplified by appropriate primer pairs, respectively. pBIG4MRScoict1 was generated by insertion of HPH into the linearized pBIG4MRSCoICT1 vector. The *coccc2*, *moict1* and *moccc2* gene replacement plasmids were generated by the procedure described above.

For the construction of the *CoICT1-mCherry* fusion gene, the *mCherry* fluorescent gene fragment and a pBIG4MRSCoICT1 vector fragment were amplified by PCR using appropriate primer pairs, respectively. pBIG4MRSCoICT1mC was generated by inserting the *mCherry* gene into the linearized pBIG4MRSCoICT1 vector.

For the construction of the plasmids used for alanine substitutions in the metal-binding site of *CoICT1*,

an approximately 0.4kb *CoICT1* fragment with designed mutations and the pBIG4MRSCoICT1 vector fragment not including the *CoICT1* ORF region (33-475 nt) were amplified by appropriate primer pairs, respectively.

Microscopic observation

For observation of infection-related morphogenesis, 20 µl conidial suspensions (1.0×10^5 or 5.0×10^5 conidia/ml) with distilled water were placed on eight spots per cover slip and incubated for 24 h at 24°C. For the observation of hyphal growth, 50 µl conidial suspensions (2.5×10^4 conidia/ml) with 0.1% yeast extract or liquid PSY medium were placed on cover glasses and incubated at 28°C for 48 h or 72 h. The conidial germination, appressoria formation and hyphal growth of each strain were observed using an Olympus BX53 microscope. Melanin pigmentation of appressoria were measured by gray scale value in ImageJ.

mCherry fluorescent signals were observed by a Leica SP8 confocal laser scanning microscope equipped with a diode-pumped solid-state 561 nm laser. Existing mCherry signals were detected from 595 to 630 nm using a SP8 hybrid detector. Images were taken using a 10× lens and a 63× oil immersion lens. The intensity of mCherry fluorescent signal in an appressorial cell was measured by imageJ.

Western blot analysis

Total protein extraction from vegetative mycelia in PSY media with or without ferimzone and $CuSO_4$ was performed as described previously (Harata and Kubo, 2014). Samples were separated by SuperSep^{TM}Ace (Wako) and transferred to polyvinylidene difluoride (PVDF) membranes. Anti-FLAG M2-peroxidase monoclonal antibody (Sigma-Aldrich) was used as a primary antibody. SuperSignal™ West Pico PLUS Chemiluminescent Substrate (Thermo Fisher) was used as a chemiluminescent substrate and signals were detected with Amersham Imager 600 imagers (GE Healthcare).

Measurement of intracellular Cu contents

Vegetative hyphae incubated for 42 h in liquid PSY media were treated with $CuSO_4$, ferimzone, or both by additional incubation for six h. After a total incubation of 48 h, mycelia were collected, washed two times with 100 μM EDTA and immediately frozen using liquid nitrogen. Freeze-dried mycelial tissues (100 mg) were digested in 60% HNO_3 at 90 °C for 140 min and H_2O_2 using DigiPREP Jr (GL SCIENCE). The Cu content of samples was measured using an iCAP 7400 Duo system (Thermo Scientific).

Pathogenicity assays

Droplet inoculations onto detached cucumber cotyledons (Cucumis sativus L. suyo) were performed using *C. orbiculare* conidia suspended with distilled water. Spray inoculations and block inoculations onto detached barley leaves (Nigrate) were performed using *M. oryzae* conidia suspended with 0.01% Tween

20 solution and mycelial blocks, respectively. Inoculated leaves were incubated in a humid box at 24°C with a 16 h photoperiod for six days.

Statistical Analysis

Error bars of the graphs represent the mean value ± standard deviation (SD). To compare two different groups, Student's t-test was used as the parametric test and Mann-Whitney U test was used as the non-parametric test. To compare two same groups, Wilcoxon signed-rank test was used as the non-parametric test. To compare multi groups, one-way analysis of variance (ANOVA) with Turkey's HSD post-hoc test was used as the parametric test and Kruskal-Wallis Test with Steel-Dwass post-hoc test as the nonparametric test. Statistical analysis was performed using Microsoft Excel 2019 and R package.

Accession Numbers

GenBank accessions: CoIct1 (TDZ25172), CoCcc2 (TDZ20317), MoIct1 (EHA56579), MoCcc2 (EHA49837), *Aspergillus fumigatus* Atx1 (EAL90217), *Saccharomyces cerevisiae* Atx1 (DAA10300), *Saccharomyces cerevisiae* Ccc2 (DAA12113).

References

Tsuji, G., Fujii, S., Fujihara, N., Hirose, C., Tsuge, S., Shiraishi, T. (2003). *Agrobacterium tumefaciens-*

mediated transformation for random insertional mutagenesis in *Colletotrichum lagenarium*. J Gen Plant Pathol. 69: 230-239.

Harata, K., and Kubo, Y. (2014). Ras GTPase activating protein CoIra1 is involved in infection-related morphogenesis by regulating cAMP and MAPK signaling pathways through CoRas2 in *Colletotrichum orbiculare*. PLoS One. 9: e109045.
